# Identification of Jak-STAT signaling involvement in sarcoidosis severity via a novel microRNA-regulated peripheral blood mononuclear cell gene signature

**DOI:** 10.1038/s41598-017-04109-6

**Published:** 2017-06-26

**Authors:** Tong Zhou, Nancy Casanova, Nima Pouladi, Ting Wang, Yves Lussier, Kenneth S. Knox, Joe G. N. Garcia

**Affiliations:** 1 Department of Physiology and Cell Biology, University of Nevada, Reno School of Medicine, Reno, NV 89557 USA; 2 Division of Pulmonary, Allergy, Critical Care, and Sleep Medicine, Department of Medicine, University of Arizona Health Sciences, Tucson, AZ 78721 USA; 3 Center for Bioinformatics and Biostatistics, University of Arizona Health Sciences, Tucson, AZ 78721 USA

## Abstract

Sarcoidosis is a granulomatous lung disorder of unknown cause. The majority of individuals with sarcoidosis spontaneously achieve full remission (uncomplicated sarcoidosis), however, ~20% of sarcoidosis-affected individuals experience progressive lung disease or cardiac and nervous system involvement (complicated sarcoidosis). We investigated peripheral blood mononuclear cell (PBMC) microRNA and protein-coding gene expression data from healthy controls and patients with uncomplicated or complicated sarcoidosis. We identified 46 microRNAs and 1,559 genes that were differentially expressed across a continuum of sarcoidosis severity (healthy control → uncomplicated sarcoidosis → complicated sarcoidosis). A total of 19 microRNA-mRNA regulatory pairs were identified within these deregulated microRNAs and mRNAs, which consisted of 17 unique protein-coding genes yielding a 17-gene signature. Pathway analysis of the 17-gene signature revealed Jak-STAT signaling pathway as the most significantly represented pathway. A severity score was assigned to each patient based on the expression of the 17-gene signature and a significant increasing trend in the severity score was observed from healthy control, to uncomplicated sarcoidosis, and finally to complicated sarcoidosis. In addition, this microRNA-regulated gene signature differentiates sarcoidosis patients from healthy controls in independent validation cohorts. Our study suggests that PBMC gene expression is useful in diagnosis of sarcoidosis.

## Introduction

Sarcoidosis remains a systemic inflammatory disease of unknown etiology with an unpredictable course that affects all races and ethnicities that is characterized by the presence of non-caseating epithelioid granulomas in one or multiple organs. The disorder is extremely heterogeneous with more than 50% of sarcoidosis patients experience remission within 3 years after diagnosis, and over 66% of patients experiencing remission within 10 years^1–3^ (uncomplicated sarcoidosis), whereas a significant percentage of patients with sarcoidosis develop granulomatous involvement of other vital organs with progressive disease (complicated sarcoidosis). Lung involvement is commonly manifested as bilateral hilar lymphadenopathy and pulmonary infiltration with more severe cases developing pulmonary fibrosis. Cardiac and neurologic involvement are also associated with significant morbidity and death^3, 4^. This broad spectrum of clinical manifestations makes diagnosis challenging and prolonged.

Clearly validated biomarkers that can accurately sub-phenotype patients with sarcoidosis are of enormous utility as they may identify subjects at increased risk of complicated sarcoidosis and allow for the delivery of targeted therapies to prevent progressive multi-organ deterioration. Unfortunately, blood, bronchoalveolar lavage fluid, exhaled breath concentrate, and cerebrospinal fluid have all been assessed as sarcoidosis biomarkers by multiple methodologies, including enzyme-linked immunosorbent assay and proteomic analysis, but with limited success. For example, angiotensin converting enzyme (ACE) is the most commonly studied biomarker but is non-specific and does not accurately correlate with severity^5, 6^. Acute phase reactants like serum amyloid (SAA) levels are significantly higher in active disease similarly to ACE^7^, but likewise suffers from low specificity and sensitivity. HLA-DR allele and cytokines such as tumor necrosis factors (TNF-α, TNF-β) are overexpressed in sarcoidosis as well and correlated with fibrosis development^8^. No single biomarker has proven to exhibit significant sensitivity and specificity to be recommended as a monitoring and prognostic tool for standard clinical use.

As a consequence, we and others have proposed the integration of blood gene expression profiling as an opportunity to explore potential useful molecular signatures in sarcoidosis^9–11^, particularly relevant in assessment of personal risk for developing complicated sarcoidosis^10^. For example, Koth *et al*. analyzed the transcriptomic gene expression data from whole blood of sarcoidosis patients and accordingly built a classifier using the gene expression data, which distinguished sarcoidosis patients from healthy controls in an external validation cohort with a fairly good accuracy^9^. Recently, we investigated genome-wide gene expression in peripheral blood mononuclear cell (PBMC) in sarcoidosis patients and identified a 20-gene signature, which distinguishes uncomplicated sarcoidosis from complicated sarcoidosis^10^. This gene signature also exhibited a substantial predictive power when classifying sarcoidosis patients from healthy controls in two external cohorts.

In this study, we proposed to incorporate microRNA (miRNA) expression information with protein-coding gene expression data to identify sarcoidosis biomarkers. Firstly, we identified a list of differentially expressed miRNAs in PBMCs from patients with sarcoidosis. Secondly, we searched for a list of target protein-coding genes for the deregulated miRNAs and based on these target genes, developed a novel miRNA-regulated peripheral blood gene signature that accurately differentiates sarcoidosis patients from healthy controls and complicated sarcoidosis patients from uncomplicated ones. Pathway analysis of the gene signature revealed Jak-STAT signaling pathway as the most significantly represented pathway. A severity score, based on the expression of the gene signature, exhibited a significantly increasing trend from healthy control, to uncomplicated sarcoidosis, and finally to complicated sarcoidosis. These studies suggest that PBMC gene expression is useful in diagnosis of sarcoidosis, and more importantly, in the identification of patients with complicated sarcoidosis.

## Results

### Identifying miRNAs differentially expressed in sarcoidosis PBMCs

To determine the differentially expressed miRNAs in sarcoidosis PBMCs, we analyzed the PBMC miRNA expression data from 35 healthy controls, 17 patients with uncomplicated sarcoidosis, and 13 patients with complicated sarcoidosis. Spearman’s rank correlation test was used to identify the miRNAs that were differentially expressed with sarcoidosis severity (healthy control → uncomplicated sarcoidosis → complicated sarcoidosis). In total, we identified 46 miRNAs (adjusted *P* < 0.05) that were differentially expressed with severity (Supplementary Table S1). Nineteen out of the 46 miRNAs showed positive correlation between expression and severity while 27 miRNAs showed negative correlation (Supplementary Table S1).

### Identifying protein-coding genes differentially expressed in sarcoidosis PBMCs

To determine the differentially expressed protein-coding genes in sarcoidosis PBMCs, we analyzed the gene expression pattern from 35 healthy controls, 17 patients with uncomplicated sarcoidosis, and 22 patients with complicated sarcoidosis (Gene Expression Omnibus [GEO]^12^ accession: GSE37912). Spearman’s rank correlation test was conducted between gene expression and sarcoidosis severity. In total, 1,559 genes showed significant correlation (adjusted *P* < 0.0005), among which 340 genes showed positive correlation between gene expression and severity while the expression of 1,219 genes was negatively correlated with severity (Supplementary Table S2).

### In silico prediction of miRNA-mRNA binding pairs

**Figure 1:**
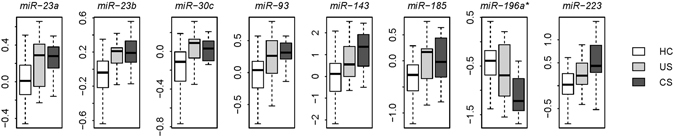
8-miRNA signature in sarcoidosis. The eight miRNAs were differentially expressed with the severity of sarcoidosis. Y-axis indicates the miRNA expression level. HC: healthy controls; US: uncomplicated sarcoidosis; CS: complicated sarcoidosis.

**Figure 2:**
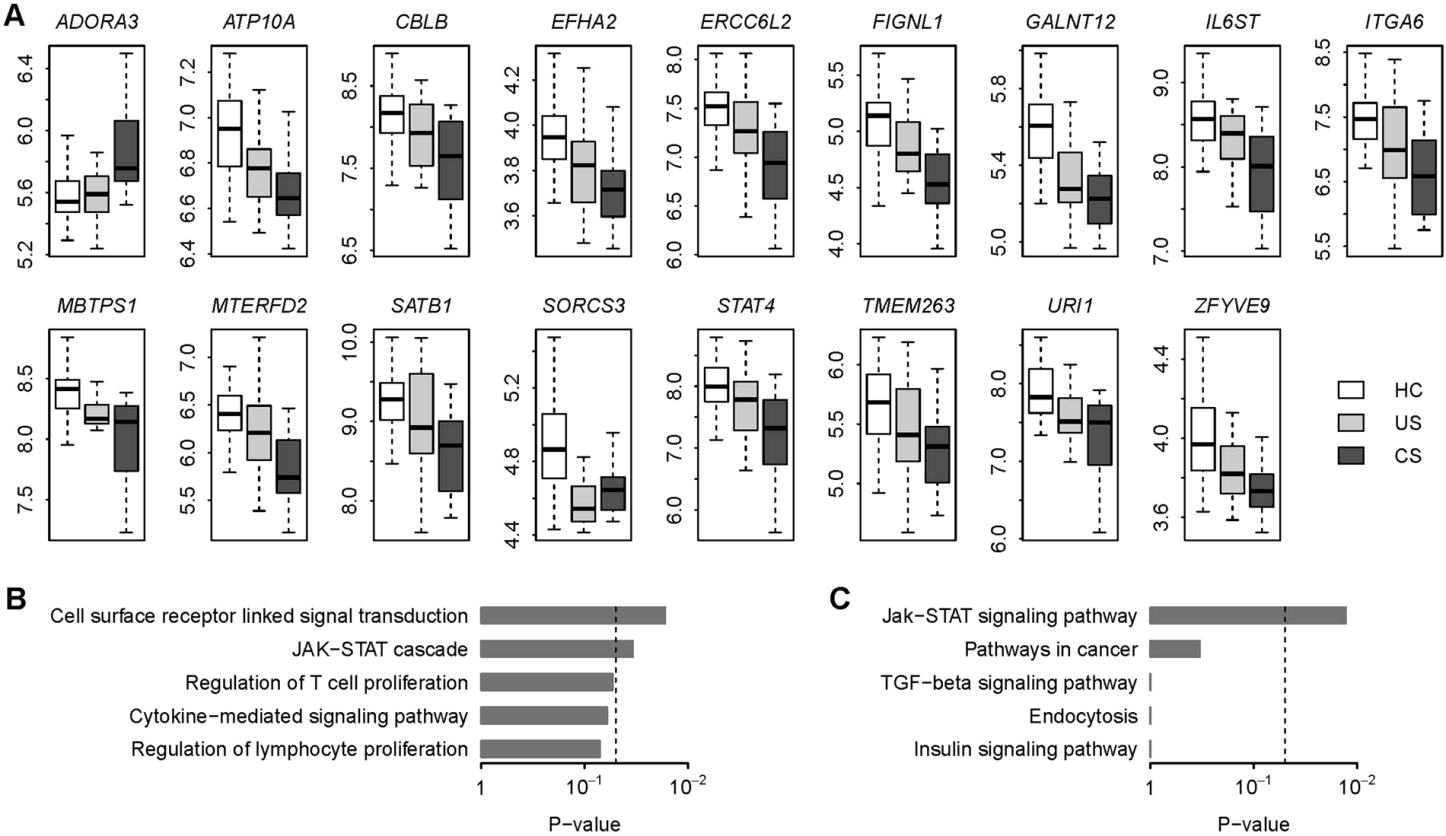
17-gene signature in sarcoidosis. (**A**) The 17 protein-coding genes were differentially expressed with the severity of sarcoidosis. Y-axis indicates the gene expression level. HC: healthy controls; US: uncomplicated sarcoidosis; CS: complicated sarcoidosis. (**B**) The top five GO biological process terms associated with the 17-gene signature. The *P*-values were calculated by Fisher’s exact test. The dash line denotes the significance level of 0.05. (**C**) The top five KEGG pathway terms associated with the 17-gene signature. The *P*-values were calculated by Fisher’s exact test. The dash line denotes the significance level of 0.05.

We searched for the target genes of the differentially expressed miRNAs, using the *in silico* prediction provided by microrna.org^13^, with filtering based on a stringent mirSVR score cutoff of −1.2 (this value represents the top 5% of mirSVR scores) to select the top miRNA-mRNA binding pairs^14^. We intersected these predicted binding targets against the differentially expressed protein-coding genes revealing a total of 425 miRNA-mRNA pairs. Among these miRNA-mRNA pairs, we only retained the pairs that demonstrated a significant negative correlation (adjusted *P* < 0.005) between miRNA and mRNA expression. This step yielded 19 miRNA-mRNA pairs consisting of eight unique miRNAs and 17 unique protein-coding genes (Table 1 and Supplementary Fig. S1). We designated the eight miRNAs as an 8-miRNA signature (Table 2), among which seven miRNAs were upregulated in complicated sarcoidosis with only one downregulated miRNA (Fig. 1). The list of 17 protein-coding genes was termed a 17-gene signature (Table 3) and contained only one gene upregulated in complicated sarcoidosis whereas the other 16 genes were downregulated (Fig. 2A). We next searched the enriched Gene Ontology (GO) biological process terms^15^ for the 17-gene signature and found that the 17-gene signature is significantly associated with “JAK-STAT cascade” (Fig. 2B). Pathway analysis based on Kyoto Encyclopedia of Genes and Genomes (KEGG)^16^ database also confirmed that the top KEGG pathway associated with the 17-gene signature is “Jak-STAT signaling pathway” (Fig. 2C).

**Table 1:** Sarcoidosis-related miRNA and target gene pairs.

miRNA	Target gene	mirSVR score	*ρ*	Adjusted *P*
*miR-23a*	*EFHA2*	−1.260	−0.589	3.42 × 10^−5^
*miR-23a*	*GALNT12*	−1.264	−0.480	1.78 × 10^−3^
*miR-23a*	*SATB1*	−1.342	−0.454	3.75 × 10^−3^
*miR-23a*	*STAT4*	−1.276	−0.475	2.12 × 10^−3^
*miR-23a*	*TMEM263*	−1.201	−0.459	3.26 × 10^−3^
*miR-23b*	*EFHA2*	−1.260	−0.531	3.49 × 10^−4^
*miR-23b*	*GALNT12*	−1.264	−0.438	5.75 × 10^−3^
*miR-30c*	*ITGA6*	−1.203	−0.433	6.46 × 10^−3^
*miR-93*	*FIGNL1*	−1.231	−0.463	2.91 × 10^−3^
*miR-93*	*MBTPS1*	−1.228	−0.533	3.23 × 10^−4^
*miR-93*	*MTERFD2*	−1.231	−0.540	2.51 × 10^−4^
*miR-93*	*URI1*	−1.273	−0.468	2.57 × 10^−3^
*miR-93*	*ZFYVE9*	−1.332	−0.511	7.03 × 10^−4^
*miR-143*	*ATP10A*	−1.333	−0.443	5.05 × 10^−3^
*miR-185*	*SORCS3*	−1.215	−0.437	5.79 × 10^−3^
miR-196a*	*ADORA3*	−1.270	−0.472	2.30 × 10^−3^
*miR-223*	*CBLB*	−1.252	−0.447	4.50 × 10^−3^
*miR-223*	*ERCC6L2*	−1.296	−0.446	4.63 × 10^−3^
*miR-223*	*IL6ST*	−1.340	−0.432	6.53 × 10^−3^

Note – *ρ* is the Spearman’s rank correlation coefficient. *P*-values were calculated by Spearman’s rank correlation test between miRNA and target gene expression levels and adjusted by Benjamini & Hochberg procedure.

**Table 2:** 8-miRNA signature in Sarcoidosis.

miRNA	*ρ*	Adjusted *P*
*miR-23a*	0.377	1.91 × 10^−2^
*miR-23b*	0.405	1.33 × 10^−2^
*miR-30c*	0.344	3.44 × 10^−2^
*miR-93*	0.386	1.78 × 10^−2^
*miR-143*	0.378	1.91 × 10^−2^
*miR-185*	0.363	2.43 × 10^−2^
miR-196a*	−0.393	1.60 × 10^−2^
*miR-223*	0.346	3.44 × 10^−2^

Note – *ρ* is the Spearman’s rank correlation coefficient. *P*-values were calculated by Spearman’s rank correlation test between miRNA expression level and sarcoidosis severity and adjusted by Benjamini & Hochberg procedure.

**Table 3:** 17-Gene Signature in Sarcoidosis.

Gene symbol	Gene title	*ρ*	Adjusted *P*
*ADORA3*	adenosine A3 receptor	0.463	3.04 × 10^−4^
*ATP10A*	ATPase, class V, type 10A	−0.541	3.58 × 10^−5^
*CBLB*	Cas-Br-M (murine) ecotropic retroviral transforming sequence b	−0.483	1.75 × 10^−4^
*EFHA2*	EF-hand domain family, member A2	−0.505	9.48 × 10^−5^
*ERCC6L2*	excision repair cross-complementation group 6-like 2	−0.508	8.92 × 10^−5^
*FIGNL1*	fidgetin-like 1	−0.565	1.77 × 10^−5^
*GALNT12*	polypeptide N-acetylgalactosaminyltransferase 12	−0.688	7.40 × 10^−8^
*IL6ST*	interleukin 6 signal transducer (gp130, oncostatin M receptor)	−0.470	2.52 × 10^−4^
*ITGA6*	integrin, alpha 6	−0.510	8.18 × 10^−5^
*MBTPS1*	membrane-bound transcription factor peptidase, site 1	−0.512	7.89 × 10^−5^
*MTERFD2*	MTERF domain containing 2	−0.533	4.31 × 10^−5^
*SATB1*	SATB homeobox 1	−0.457	3.65 × 10^−4^
*SORCS3*	sortilin-related VPS10 domain containing receptor 3	−0.457	3.62 × 10^−4^
*STAT4*	signal transducer and activator of transcription 4	−0.500	1.11 × 10^−4^
*TMEM263*	transmembrane protein 263	−0.463	3.06 × 10^−4^
*URI1*	URI1, prefoldin-like chaperone	−0.503	1.01 × 10^−4^
*ZFYVE9*	zinc finger, FYVE domain containing 9	−0.478	2.01 × 10^−4^

Note – *ρ* is the Spearman’s rank correlation coefficient. *P*-values were calculated by Spearman’s rank correlation test between protein-coding gene expression level and sarcoidosis severity and adjusted by Benjamini & Hochberg procedure.

### Classification power of the 8-miRNA signature

We first tested the classification power of the 8-miRNA signature in our discovery cohort. A severity score (*S*_*miRNA*_) was assigned to each patient based on the expression of the 8-miRNA signature, which is a linear combination of the miRNA expression values weighted by direction of differential expression (See Methods for details). Higher *S*_*miRNA*_ implies more severe sarcoidosis. As expected, a significant positive correlation (Spearman’s rank correlation test: *ρ* = 0.651 and *P* = 4.4 × 10^−9^) was identified between *S*_*miRNA*_ and sarcoidosis severity in our discovery cohort (Supplementary Fig. S2).

**Figure 3:**
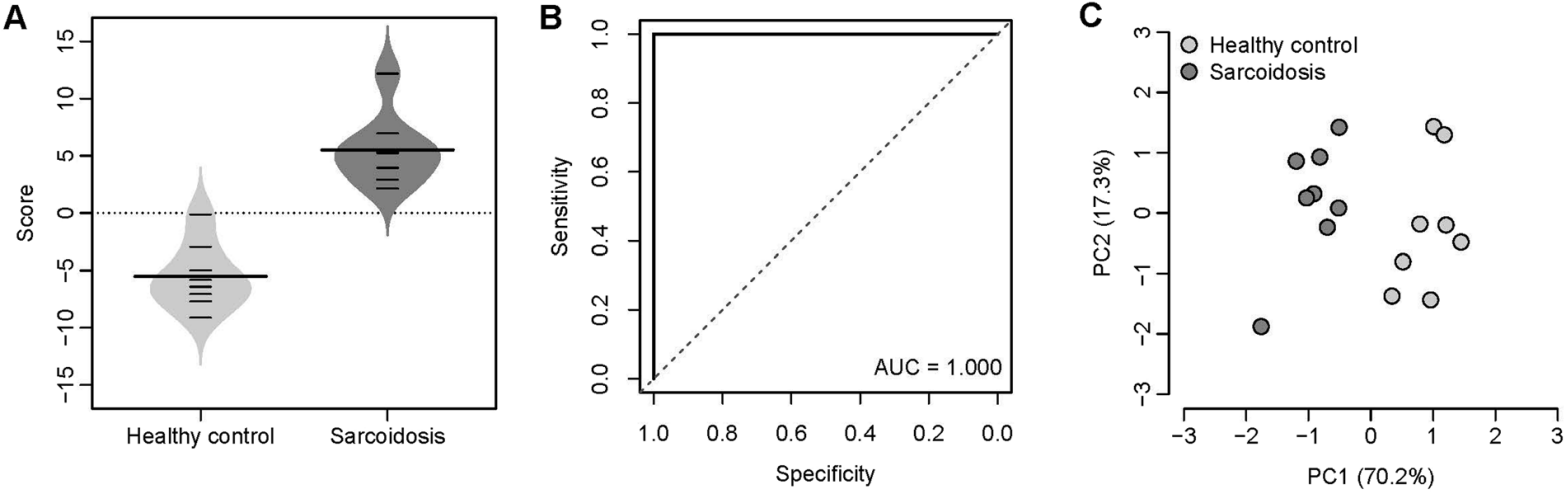
The performance of the 8-miRNA signature in the sarcoidosis validation cohort. (**A**) The 8-miRNA signature based severity score differentiates the sarcoidosis patients from the healthy controls in the Berlin cohort. The violin plot indicates the distribution of the severity score in each category. (**B**) The ROC curve of the 8-miRNA signature in classifying the subjects in the Berlin cohort. The AUC is equal to one. (**C**) Principal component analysis on the expression of the 8-miRNA signature. X-axis: the first principal component with eigenvalue; Y-axis: the second principal component with eigenvalue.

We next investigated the classification power of the 8-miRNA signature in a validation dataset from Germany (GEO accession: GSE34608)^17^, which contains the whole blood gene expression data for eight healthy controls and eight patients with sarcoidosis (Germany cohort). The 8-miRNA signature based severity score of sarcoidosis patients is significantly higher than that of controls in the Germany cohort (t-test: *P* = 3.4 × 10^−6^) (Fig. 3A). The areas under the receiver operating characteristic (ROC) curve (*AUC*) is 1.000 in this cohort (Fig. 3B), which suggests a fairly good classification accuracy of the 8-miRNA signature. Principal component analysis on the 8-miRNA expression also indicates that the sarcoidosis patients can be clearly distinguished from the health controls (Fig. 3C).

### Classification power of the 17-gene signature

Similar to the strategy employed for the 8-miRNA signature, we assigned a severity score (*S*_*gene*_) to each patient based on the expression of the 17-gene signature, which is a linear combination of the protein-coding gene expression values weighted by direction of differential expression (see Methods for details). Patients with more severe sarcoidosis are expected to have a higher *S*_*gene*_. Not surprisingly, we found a significant positive correlation (Spearman’s rank correlation test: *ρ* = 0.615 and *P* = 5.6 × 10^−9^) between *S*_*gene*_ and sarcoidosis severity in our discovery cohort (Supplementary Fig. S3).

**Figure 4:**
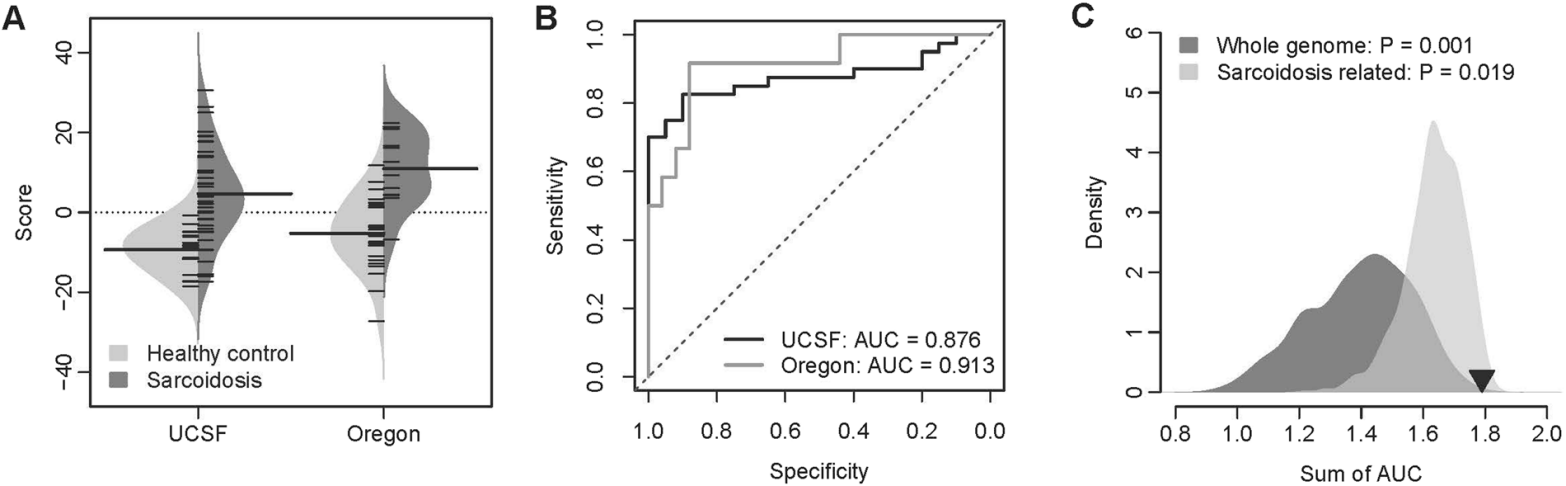
The performance of the 17-gene signature in the validation cohorts. (**A**) The 17-gene signature based severity score differentiates the sarcoidosis patients from the healthy controls in the UCSF and Oregon cohorts. The violin plot indicates the distribution of the severity score in each category. (**B**) The ROC curves of the 17-gene signature in classifying the subjects in the UCSF and Oregon cohorts. (**C**) Superior predictive power of the 17-gene signature compared with random gene set. The dark grey area shows the distribution of the sum of AUC (both the validation cohorts) for the 1,000 resampled gene signatures (with the identical size as the 17-gene signature) randomly picked up from human genome. The light grey area shows the distribution of the sum of AUC for the 1,000 resampled gene signatures randomly selected from the pool of the sarcoidosis related genes. The black triangle stands for the sum of AUC of the 17-gene signature. Right-tailed *P*-values of the sampling distribution were calculated.

We next validated the predictive power of the 17-gene signature in two independent blood gene expression datasets. One dataset (GEO accession: GSE19314)^9^ is from University of California, San Francisco that contains 20 healthy controls and 40 sarcoidosis patients (UCSF cohort). The other dataset (GEO accession: GSE18781)^18^ is from Oregon Health Sciences University and includes 25 healthy controls and 12 sarcoidosis patients (Oregon cohort). The 17-gene signature-based severity score of sarcoidosis patients is significantly higher than that of controls in both the validation cohorts (t-test: *P* = 1.5 × 10^−8^ for the UCSF cohort and *P* = 3.8 × 10^−5^ for the Oregon cohort) (Fig. 4A). The *AUC* values of classification are 0.876 and 0.913 in the UCSF and Oregon cohorts, respectively (Fig. 4B). Principal component analysis on the expression of the 17-gene signature indicates that the sarcoidosis patients can be well distinguished from the health controls (Supplementary Fig. S4).

To understand the discriminative power of the 17-gene signature in distinguishing sarcoidosis from other lung diseases, we collected two more blood transcriptomic datasets (GEO accession: GSE42826 and GSE4257), the first consisted of 52 healthy controls, 25 sarcoidosis patients, 11 tuberculosis (TB) patients, six pneumonia patients, and eight lung cancer patients. TB, pneumonia patients, lung cancer were considered as non-sarcoidosis lung disorders. The second dataset comprised of 123 healthy controls, 87 and 47 cases with COPD and sarcoidosis respectively. Principal component analysis on the expression of the 17-gene signature in this dataset indicates that the first principal component significantly differentiates the sarcoidosis patients from the non-sarcoidosis lung disorders in both datasets (t-test: *P* = 1.9 × 10^−2^ and 2.09 × 10^−5^) (Supplementary Figs S5 and S6), which suggests that the 17-gene signature is able to distinguish between sarcoidosis and non-sarcoidosis lung disorders. To compare the effect of systemic corticosteroids on our 17-gene signature, we also surveyed a gene expression data set (GSE67255) on 18 healthy subjects before and 24 hours after the administration of glucocorticoids^19^. The differential expression was performed using one-way repeated measure ANOVA. None of the 17 genes in our signature were significantly affected (*P* < 0.5).

A computational study by Venet *et al*. suggests that most published gene signatures are not significantly better than random gene sets of identical size that are randomly picked up from human genome^20^. To address this issue, resampling test was applied. We obtained 1,000 random gene signatures by randomly selecting 17 genes from human genome. For each random gene set, we calculated the *AUC* values for both the UCSF and Oregon cohorts. The sum of *AUC* in both cohorts was recorded for each random gene signature, which measured the classification power of the random gene set. Our alternative hypothesis is that the sum of *AUC* of our 17-gene signature should be more positive than expected by chance if the classification power of the 17-gene signature is significantly better than the random gene signatures. By resampling test, we found that we could reject the null hypothesis that the classification power of the 17-gene signature is by chance. The sum of *AUC* of our 17-gene signature is significantly larger than that of random gene signatures (Right-tailed: *P* = 0.001) (Fig. 4C).

We next addressed whether the classification power of the 17-gene signature is superior to the other genes that are related to sarcoidosis. To answer this question, we conducted a second resampling test. We limited the resampling pool to the genes that were differentially expressed with sarcoidosis severity (1,559 genes listed in Supplementary Table S2) and defined these genes as sarcoidosis related. We then randomly selected 17 genes from the pool of sarcoidosis related genes and tested the predictive power of the random gene signature. We repeated this randomization procedure for 1,000 times. The performance of the random gene signature was quantified by the sum of *AUC* in both validation cohorts. Figure 4C demonstrates that the classification power of the 17-gene signature is significantly better than that of 1,000 resampled sarcoidosis related gene signatures (Right-tailed: *P* = 0.019).

## Discussion

Sarcoidosis remains a multifactorial, complex, and challenging systemic disease, whose unpredictable course mandates the urgent application of novel approaches, such as genome-based expression profiling, to generate personalized risk assessment tools to diagnose, monitor disease progression, and guide therapeutic management of those with this affliction. Here, we stratified sarcoidosis cases into two different categories: uncomplicated and complicated sarcoidosis. Uncomplicated cases presented remission, while complicated cases exhibited significant organ impairment. The accurate identification of patients at risk for complicated sarcoidosis is a clinical challenge. Biomarkers or molecular signatures emerging from new technologies such as PBMC miRNA/gene expression analysis represent an opportunity to change the routine clinical care of sarcoidosis. In this study, we investigated the PBMC miRNA and protein-coding gene expression data from both healthy controls and patients with sarcoidosis. Using these data, we identified a molecular signature consisting of 17 protein-coding genes for diagnostic purpose in sarcoidosis. A severity score was assigned to each human subject based on the expression of the 17-gene signature. A higher score suggested a higher likelihood of complicated sarcoidosis. In our discovery cohort, we observed a significant increasing trend in the severity score from healthy control, uncomplicated sarcoidosis, to complicated sarcoidosis. We also demonstrated that this miRNA-regulated gene signature can differentiate sarcoidosis patients from healthy controls in independent validation cohorts.

We initially identified a 8-miRNA signature (7 upregulated and 1 downregulated miRNAs in complicated sarcoidosis) that potentially relate to sarcoidosis severity, including miR-23a, miR-23b, miR-30c, miR-185, and miR-223, now recognized to be related to pulmonary hypertension^21–25^ and lung cancer^26–29^. In a previous study, PBMC miRNA expression profiles were compared between sarcoidosis patients and healthy controls^30^. However, we failed to identify any single miRNA within our 8-miRNA signature that overlapped with the reported list of differentially expressed miRNAs^30^. This lack of overlap may reflect the difference in methodology and objective between the two studies: we mainly focused on complicated sarcoidosis and correlation test was applied to find the miRNAs associated with sarcoidosis severity. Based on the expression of the 8-miRNA signature, we developed a scoring system. This severity score reflected the extent and severity of sarcoidosis. We demonstrated that this miRNA-based score differentiates sarcoidosis patients in both discovery and validation cohorts.

We also explored the utility of a protein-coding gene expression signature in sarcoidosis. We identified the 17-gene signature, in which the genes were potentially regulated by the miRNAs within the 8-miRNA signature. In agreement with prior reported studies, the 17-gene signature was enriched in Jak-STAT signaling pathway^9, 10, 18^. Jak-STAT signaling pathway is an intracellular cascade initiated in response to cytokine signaling. Several genes in Jak-STAT pathways have already been associated with sarcoidosis, such as *IL15* ^31^, *IL23R* ^32^, and *STAT1* ^18^. According to the current knowledge of the molecular mechanism leading to sarcoidosis, genes involved in T-cell receptor selection, T-cell activation and apoptosis, and cytokine regulation are also highly associated with the pathogenesis of sarcoidosis^9, 10, 33^. In our 17-gene signature, *CBLB* and *IL6ST* are known to be involved in T-cell receptor signaling and cytokine-cytokine receptor interaction, respectively, according to the definition in KEGG database. However, we failed to identify a single gene within our 17-gene signature that overlapped with the previously published sarcoidosis blood gene signatures by Koth *et al*.^9^ and Zhou *et al*.^10^, respectively.

A published bioinformatical study by Venet *et al*. suggests that resampling test should be used to evaluate the predictive power of a given gene signature instead of focusing on nominal *P*-value, because most the published signatures are not significantly better than the randomized gene signatures of identical size^20^. Using resampling tests, we found that the classification power of the 17-gene signature is significantly better than that of the random gene sets selected from human genome.

In contrast to the published sarcoidosis gene signatures derived from whole genome screening by Koth *et al*.^9^ and Zhou *et al*.^10^, the 17-gene signature was developed based on miRNA expression information and predefined miRNA-gene interactions. Statistically-derived gene signatures by whole genome screening are often highly accurate in the patient data sets from which they were identified, yet most of them have not been validated as useful clinical tools. Using resampling test, we demonstrate that the miRNA-regulated 17-gene signature performs even better than the random gene sets selected from the pool of sarcoidosis related genes, which suggests that incorporation miRNA expression information into gene signature identification may significantly decrease false positive rate when developing gene signatures for complex human diseases.

Despite multiple efforts to identify concordant DE-miRNA in sarcoidosis, it is recognized that patterns of miRNA expression differ between BAL cells, lung tissue, lymph nodes and PBMCs suggesting organ specific regulation. In our initial 8-miRNa signature we identified miR-23a, miR-23b, miR-30c, miR-185, and miR-223 related to sarcoidosis severity. Kiszalkiewicz *et al*. recently published overexpression of miRNas involved in angiogenesis, miRNA -27b, miR-192 and miR-221 in BALF cells from patients with acute sarcoidosis, none of these were significantly overexpressed in PBMCs^34^. Crouser *et al*. also profiled DE-miRNA in lung and lymph tissue and observed no overlap in miRNA expression in PBMC relative to diseased lung tissue; however TGF Fβ/WNT was a commonly regulated pathway^30^. Jazwa *et al*., suggested miRNA-34 regulates *SIRT1* and the expression of IFN- γ in sarcoidosis^34, 35^.

In summary, we derived a molecular signature consisting of 17 protein-coding genes, which are potentially regulated by deregulated miRNA in sarcoidosis. This signature can be independently used as potential novel molecular markers for differentiating patients with sarcoidosis, especially for distinguishing the patients with risk of complicated sarcoidosis. Although gene expression variation in PBMCs may not necessarily reflect the dynamics of the tissue microenvironment in sarcoidosis patients, PBMC gene expression information is useful in diagnosis of sarcoidosis, and more importantly, in the identification of patients with complicated sarcoidosis.

## Methods

### PBMC samples

The study was approved by the University of Arizona Institutional Review Board with written informed consent obtained from all subjects, and was performed in accordance with the principles in the Declaration of Helsinki. We defined complicated sarcoidosis as cardiac sarcoidosis (e.g. ventricular arrhythmias)^36^, neurologic sarcoidosis (e.g. evidence of hyperdense MRI lesions)^37^, or severe pulmonary sarcoidosis (forced vital capacity < 50%) (Supplementary Table S3). The diagnosis of sarcoidosis was based on established joint international criteria^38^. For our discovery cohort, PBMC samples were collected from 35 healthy controls and 39 sarcoidosis patients (17 patients with uncomplicated sarcoidosis and 22 patients with complicated sarcoidosis). The healthy controls were collected in a matching procedure for both age and gender of the patients. As for the sarcoidosis patients, we didn’t find significant difference in age (t-test: P > 0.05) and gender (χ2-test: P > 0.05) between uncomplicated and complicated cases. Patient characteristics and treatment information are summarized in Supplementary Table S4.

### High-throughput miRNA expression data

For the discovery cohort, we profiled the PBMC miRNA expression level for 35 healthy controls, 17 patients with uncomplicated sarcoidosis, and 13 patients with complicated sarcoidosis, using Exiqon miRCURY LNA Array v11.0 (Exiqon, Inc., Denmark). Total RNA was isolated from PBMCs according to manufacturer’s protocol. Array hybridization was performed by Exiqon with the quantified signals background corrected using *normexp* with offset value 10 based on a convolution model^39^ and normalized using the global Lowess regression algorithm. Only the miRNAs being present in at least two third of total samples were further analyzed. The miRNA expression data of the validation cohort, the Germany cohort, were obtained from the GEO database (GEO accession: GSE34608), which were based on Agilent-019118 Human miRNA Microarray 2.0 G4470B.

### High-throughput protein-coding gene expression data

The protein-coding gene expression data of the discovery cohort are available from the GEO database (GEO accession: GSE37912)^10^, which were based on Affymetrix Human Exon 1.0ST Array. Briefly, gene expression data were summarized using robust multi-array average (RMA) algorithm^40^ embedded in the Affymetrix Power Tools v.1.12.0. Adjustment for possible batch effect was conducted by COMBAT^41^. We consider a transcript cluster to be reliably expressed in these samples if the Affymetrix implemented DABG (detection above ground)^42^ *P*-value was less than 0.01 in at least two third of total samples. The gene expression data of the UCSF (GEO accession: GSE19314)^9^ and Oregon cohorts (GEO accession: GSE18781)^18^ were downloaded from the GEO database, which were based on Affymetrix Human Genome U133 Plus 2.0 Array.

### Severity score

8-miRNA and 17-gene based severity scores were calculated for each human subject, respectively. 8-miRNA based severity score (*S*_*miRNA*_) is a linear combination of expression values of the miRNAs within the 8-miRNA signature. The formula is shown below:

1 $${{S}}_{{miRNA}}=\sum _{i=1}^{{N}_{miRNA}}{\rm{sgn}}({{\rho }}_{{i}}^{{miRNA}})({{e}}_{{i}}^{{miRNA}}-{{\mu }}_{{i}}^{{miRNA}})/{{\tau }}_{{i}}^{{miRNA}}$$

Here, *N*_*miRNA*_ is the number of miRNAs in the 8-miRNA signature; ${\rho }_{i}^{miRNA}$ is the Spearman’s rank correlation coefficient of miRNA *i* (as shown in Table 2); ${{e}}_{{i}}^{{miRNA}}$ is the expression level of miRNA *i*; ${\mu }_{i}^{miRNA}$ and ${\tau }_{i}^{miR{NA}}$ are the mean and standard deviation of the expression values for m*i*RNA *i* across all samples, respectively; and “sgn” denotes the sign function. 17-gene based severity score (*S*_*gene*_) is a linear combination of expression values of the genes within the 17-gene signature^43–45^. The formula is shown below:

2 $${{S}}_{{gene}}=\sum _{i=1}^{{N}_{gene}}{\rm{sgn}}({{\rho }}_{{i}}^{{gene}})({{e}}_{{i}}^{{gene}}-{{\mu }}_{{i}}^{{gene}})/{{\tau }}_{{i}}^{{gene}}$$

Here, *N*_*gene*_ is the number of protein-coding genes in the 17-gene signature; ${{\rho }}_{{i}}^{{gene}}$ is the Spearman’s rank correlation coefficient of gene *i* (as shown in Table 3); ${{e}}_{{i}}^{{gene}}$ is the expression level of gene *i*; ${{\mu }}_{{i}}^{{gene}}$ and ${{\tau }}_{{i}}^{{gene}}$ are the mean and standard deviation of the expression values for gene *i* across all samples, respectively; and “sgn” denotes the sign function.

## Electronic supplementary material

Supplementary Information
