# Supplementary material for: Identification of Jak-STAT signaling involvement in sarcoidosis severity via a novel microRNA-regulated peripheral blood mononuclear cell gene signature
Source: Sci Rep. 2017 Jun 26;7:4237. doi: 10.1038/s41598-017-04109-6 (PMC5484682; doi:10.1038/s41598-017-04109-6)
Supplement: Supplementary file 1 — Supplementary Information [file 41598_2017_4109_MOESM1_ESM.pdf]

Supplementary information of "Identification of Jak-STAT signaling involvement in sarcoidosis severity via a novel microRNA-regulated peripheral blood mononuclear cell gene signature"

Tong Zhou[1†]
Nancy Casanova[2†]
Nima Pouladi[3]
Ting Wang[2]
Yves Lussier[3]
Kenneth S. Knox[2]
Joe G.N. Garcia[2*]

[1]Department of Physiology and Cell Biology, University of Nevada School of Medicine, Reno, NV 89557, USA
[2]Division of Pulmonary, Allergy, Critical Care, and Sleep Medicine, Department of Medicine, University of Arizona, Tucson, AZ 78721, USA
[3]Center for Bioinformatics and Biostatistics, University of Arizona Health Sciences, Tucson, AZ, 78721, USA

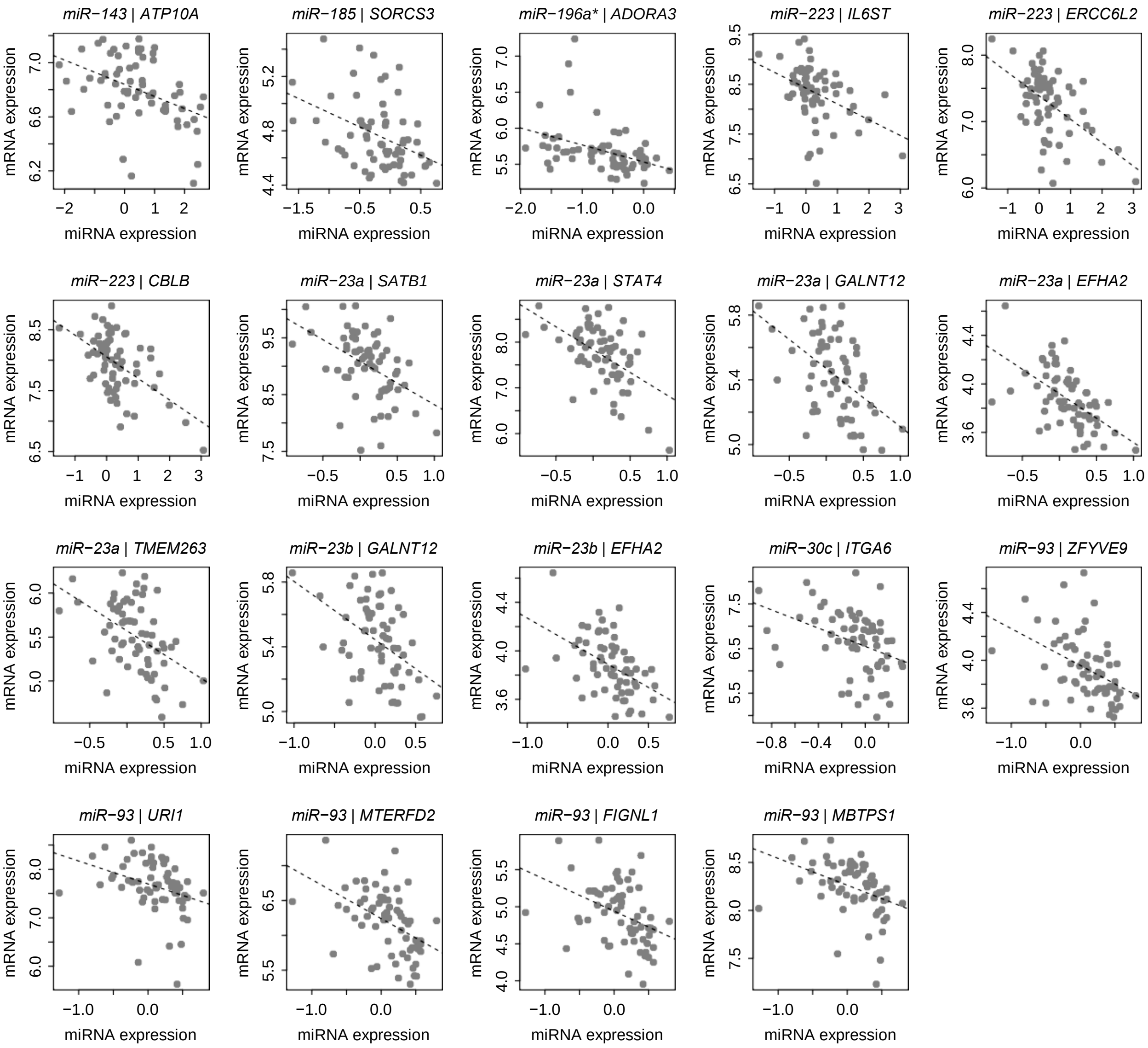

Figure S1. The negative correlation between miRNA and gene expression for the 19 miRNA-gene pairs. The 19 miRNA-gene pairs consist eight unique miRNAs (8-miRNA signature) and 17 unique protein-coding genes (17-gene signature). X-axis: miRNA expression level; Y-axis: gene expression level.

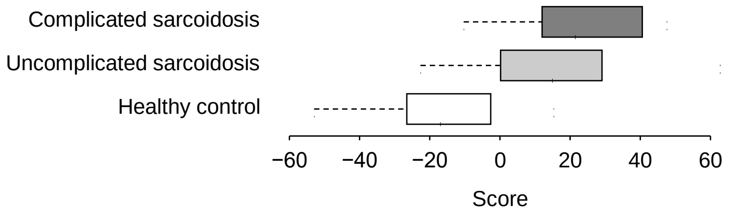

Figure S2. The 8-miRNA signature based severity score differentiates the subjects in the discovery cohort.

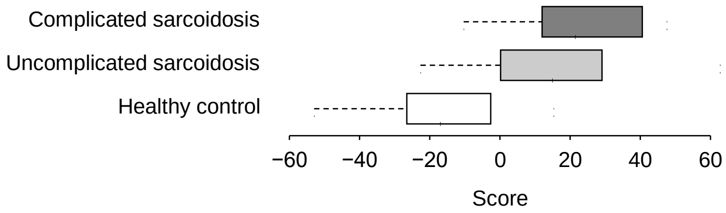

Figure S3. The 17-gene signature based severity score differentiates the subjects in the discovery cohort.

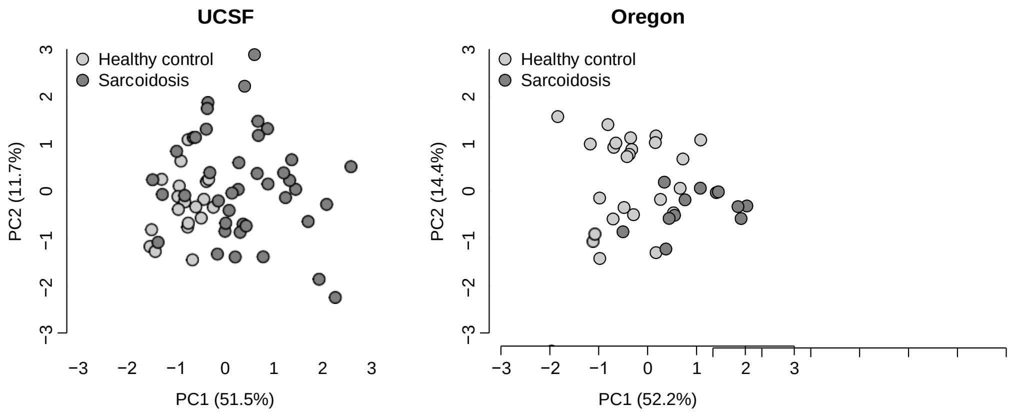

Figure S4. Principal component analysis on the expression of the 17-gene signature in the validation cohorts. X-axis: the first principal component with eigenvalue; Y-axis: the second principal component with eigenvalue.

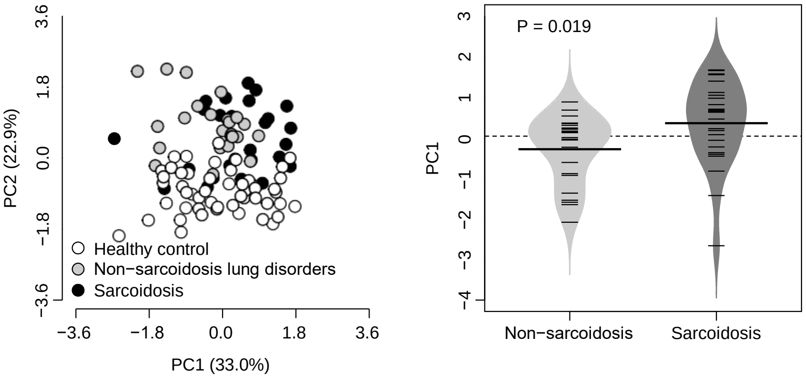

Figure S5. The 17-gene signature distinguishes between sarcoidosis and non-sarcoidosis lung disorders. Left panel: principal component analysis on the expression of the 17-gene signature in the London cohort. X-axis: the first principal component with eigenvalue; Y-axis: the second principal component with eigenvalue. Right panel: the first principal component (PC1) distinguishes between sarcoidosis and non-sarcoidosis lung disorders. The P -value was computed by t-test.

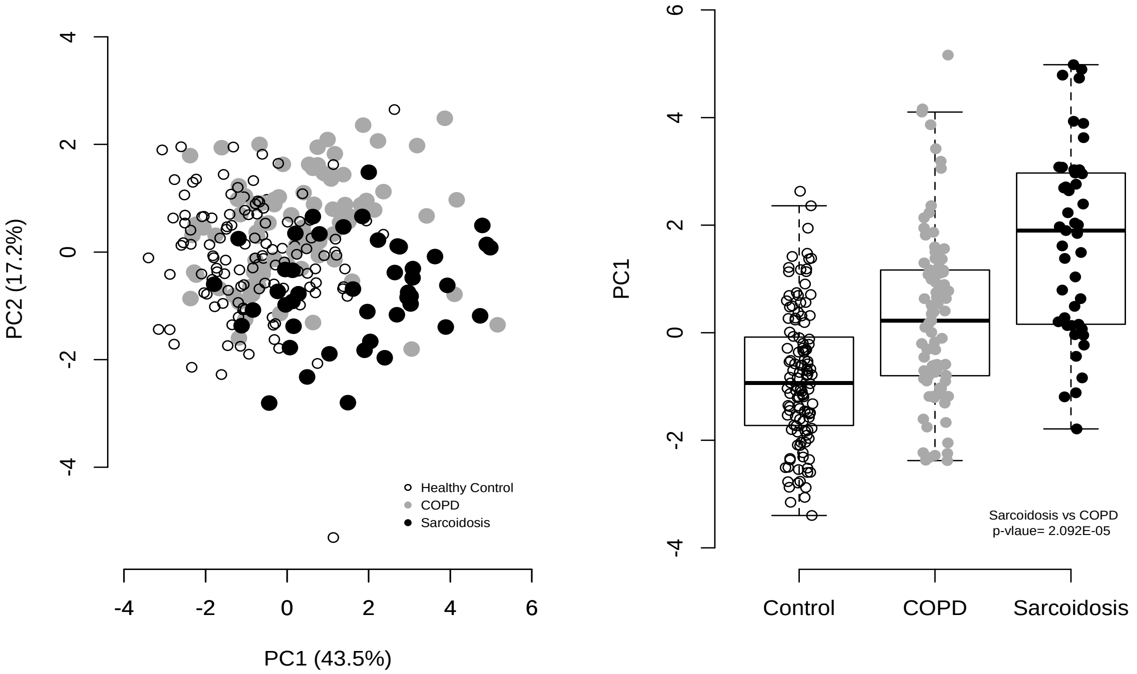

Figure S6. The 17-gene signature identifies the patients with sarcoidosis from those with COPD. Left panel: principal component analysis was performed on the expression values of the 17-gene signature after merging COPD (GSE42057) and London cohorts through the use of COMBAT. The merged dataset is comprised of 123 healthy controls, 87 and 45 cases with COPD and sarcoidosis, respectively. X-axis and Y-axis are the first and the second principal components, respectively, with the percentages of variance they explain. Right panel: the first principal component (PC1) significantly separates sarcoidosis and COPD cases (t-test p-value= 2.09E-05).

Table S1. The miRNAs that are differentially expressed with sarcoidosis severity

miRNA	ρ	Adjusted P
*miR-888**	-0.543	7.24E-4
miR-494	-0.497	2.46E-3
miR-150	-0.476	3.88E-3
miR-342-3p	-0.455	7.08E-3
miR-549	-0.440	9.46E-3
miR-720	-0.436	9.46E-3
*miR-181a-2**	-0.434	9.46E-3
miR-1248	-0.424	1.09E-2
miR-208a	-0.410	1.33E-2
miR-519d	-0.404	1.33E-2
miR-1280	-0.401	1.33E-2
miR-589	-0.400	1.33E-2
*miR-196a**	-0.393	1.60E-2
miR-412	-0.390	1.66E-2
miR-491-3p	-0.383	1.78E-2
miR-550	-0.380	1.89E-2
miR-548e	-0.373	2.04E-2
miR-1260	-0.370	2.13E-2
miR-342-5p	-0.364	2.43E-2
miR-1274a	-0.362	2.43E-2
miR-1264	-0.358	2.70E-2
miR-1274b	-0.343	3.44E-2
*miR-149**	-0.342	3.47E-2
miR-891a	-0.340	3.55E-2
miR-874	-0.335	3.84E-2
miR-634	-0.335	3.84E-2
miR-31	-0.326	4.53E-2
miR-484	0.322	4.81E-2
miR-107	0.325	4.53E-2
miR-17	0.333	3.89E-2
miR-744	0.337	3.74E-2
miR-24	0.344	3.44E-2
miR-30c	0.344	3.44E-2
miR-191	0.344	3.44E-2
miR-223	0.346	3.44E-2
miR-185	0.363	2.43E-2
miR-23a	0.377	1.91E-2
miR-143	0.378	1.91E-2
miR-199a-5p	0.383	1.78E-2
miR-93	0.386	1.78E-2
miR-23b	0.405	1.33E-2

miRNA	ρ	Adjusted *P*
*miR-425**	0.406	1.33E-2
miR-145	0.410	1.33E-2
miR-425	0.414	1.33E-2
miR-103	0.431	9.46E-3
miR-326	0.494	2.46E-3

Note – ρ is the Spearman's rank correlation coefficient. *P*-values were calculated by Spearman's rank correlation test between miRNA expression level and sarcoidosis severity and adjusted by Benjamini & Hochberg procedure.

Table S2. The protein-coding genes that are differentially expressed with sarcoidosis severity

Gene symbol	Gene title	ρ	Adjusted *P*
MEI1	meiosis inhibitor 1	-0.701	3.86E-8
GALNT12	polypeptide N-acetylgalactosaminyltransferase 12	-0.688	7.40E-8
TSHZ2	teashirt zinc finger homeobox 2	-0.664	3.20E-7
ZNF30	zinc finger protein 30	-0.664	3.20E-7
DENND2D	DENN/MADD domain containing 2D	-0.648	1.01E-6
ZNF671	zinc finger protein 671	-0.644	1.14E-6
KLHL28	kelch-like 28 (Drosophila)	-0.637	1.36E-6
ITPKB	inositol 1,4,5-trisphosphate 3-kinase B	-0.635	1.36E-6
KBTBD3	kelch repeat and BTB (POZ) domain containing 3	-0.634	1.36E-6
TP53BP1	tumor protein p53 binding protein 1	-0.633	1.36E-6
NFATC2	nuclear factor of activated T-cells, cytoplasmic, calcineurin-dependent 2	-0.632	1.36E-6
MSL2	male-specific lethal 2 homolog (Drosophila)	-0.632	1.36E-6
TRIM68	tripartite motif-containing 68	-0.632	1.36E-6
ZNF227	zinc finger protein 227	-0.620	3.09E-6
CSRNP2	cysteine-serine-rich nuclear protein 2	-0.619	3.17E-6
RBM12B	RNA binding motif protein 12B	-0.617	3.25E-6
SLC30A4	solute carrier family 30 (zinc transporter), member 4	-0.613	3.56E-6
TNRC6C	trinucleotide repeat containing 6C	-0.613	3.56E-6
SLAIN1	SLAIN motif family, member 1	-0.612	3.56E-6
ZNF397	zinc finger protein 397	-0.612	3.56E-6
ZFP3	zinc finger protein 3 homolog (mouse)	-0.611	3.72E-6
GCET2	germinal center expressed transcript 2	-0.610	3.78E-6
CERK	ceramide kinase	-0.609	3.78E-6
ZNF570	zinc finger protein 570	-0.607	3.78E-6
PLEKHA1	pleckstrin homology domain containing, family A (phosphoinositide binding specific) member 1	-0.607	3.78E-6
MAGOHB	mago-nashi homolog B (Drosophila)	-0.607	3.78E-6
SCAND2	SCAN domain containing 2 pseudogene	-0.607	3.78E-6
ZNF540	zinc finger protein 540	-0.607	3.78E-6
ASB1	ankyrin repeat and SOCS box-containing 1	-0.605	4.17E-6
LAX1	lymphocyte transmembrane adaptor 1	-0.604	4.29E-6
SACS	spastic ataxia of Charlevoix-Saguenay (sacsin)	-0.602	4.74E-6
LMO7	LIM domain 7	-0.601	4.94E-6
TTC3	tetratricopeptide repeat domain 3	-0.599	5.62E-6
RECK	reversion-inducing-cysteine-rich protein with kazal motifs	-0.598	5.63E-6
CD247	CD247 molecule	-0.598	5.63E-6
NCOA5	nuclear receptor coactivator 5	-0.597	5.72E-6
KIF3A	kinesin family member 3A	-0.596	6.25E-6
NR3C2	nuclear receptor subfamily 3, group C, member 2	-0.595	6.35E-6
COQ10A	coenzyme Q10 homolog A (S. cerevisiae)	-0.592	7.65E-6
STK17A	serine/threonine kinase 17a	-0.590	8.27E-6
C3orf31	chromosome 3 open reading frame 31	-0.589	8.55E-6
EML5	echinoderm microtubule associated protein like 5	-0.588	9.20E-6
TRIM4	tripartite motif-containing 4	-0.586	1.02E-5
DDX55	DEAD (Asp-Glu-Ala-Asp) box polypeptide 55	-0.584	1.11E-5
HLTF	helicase-like transcription factor	-0.583	1.19E-5

Gene symbol	Gene title	ρ	Adjusted P
ADAM20	ADAM metallopeptidase domain 20	-0.582	1.19E-5
FANCF	Fanconi anemia, complementation group F	-0.582	1.19E-5
DYRK2	dual-specificity tyrosine-(Y)-phosphorylation regulated kinase 2	-0.582	1.19E-5
TTC12	tetratricopeptide repeat domain 12	-0.581	1.20E-5
TTLL5	tubulin tyrosine ligase-like family, member 5	-0.580	1.26E-5
C12orf26	chromosome 12 open reading frame 26	-0.580	1.26E-5
PRKCA	protein kinase C, alpha	-0.580	1.26E-5
WDR52	WD repeat domain 52	-0.580	1.26E-5
KIAA1279	KIAA1279	-0.578	1.34E-5
SCAI	suppressor of cancer cell invasion	-0.578	1.35E-5
SIDT1	SID1 transmembrane family, member 1	-0.577	1.40E-5
TEX10	testis expressed 10	-0.576	1.45E-5
PARP2	poly (ADP-ribose) polymerase 2	-0.576	1.45E-5
ST6GAL1	ST6 beta-galactosamide alpha-2,6-sialyltranferase 1	-0.575	1.47E-5
ZNF597	zinc finger protein 597	-0.575	1.47E-5
PSIP1	PC4 and SFRS1 interacting protein 1	-0.575	1.47E-5
KIAA1328	KIAA1328	-0.574	1.47E-5
HELQ	helicase, POLQ-like	-0.574	1.47E-5
ZNF568	zinc finger protein 568	-0.574	1.47E-5
C2CD3	C2 calcium-dependent domain containing 3	-0.573	1.47E-5
CDKN2AIP	CDKN2A interacting protein	-0.573	1.47E-5
FAM118A	family with sequence similarity 118, member A	-0.573	1.47E-5
C2orf42	chromosome 2 open reading frame 42	-0.573	1.47E-5
ING4	inhibitor of growth family, member 4	-0.573	1.47E-5
SLC6A16	solute carrier family 6, member 16	-0.572	1.51E-5
NPC1	Niemann-Pick disease, type C1	-0.572	1.51E-5
PIP4K2B	phosphatidylinositol-5-phosphate 4-kinase, type II, beta	-0.572	1.51E-5
NPHP3	nephronophthisis 3 (adolescent)	-0.572	1.51E-5
PAFAH2	platelet-activating factor acetylhydrolase 2, 40kDa	-0.571	1.55E-5
LBH	limb bud and heart development homolog (mouse)	-0.571	1.55E-5
TP53RK	TP53 regulating kinase	-0.571	1.55E-5
C1orf103	chromosome 1 open reading frame 103	-0.570	1.60E-5
ZNF287	zinc finger protein 287	-0.570	1.60E-5
PHF17	PHD finger protein 17	-0.569	1.60E-5
INADL	InaD-like (Drosophila)	-0.569	1.60E-5
KLRAP1	killer cell lectin-like receptor subfamily A pseudogene 1	-0.569	1.60E-5
ZNF614	zinc finger protein 614	-0.569	1.62E-5
ZNF84	zinc finger protein 84	-0.568	1.65E-5
STK38	serine/threonine kinase 38	-0.568	1.67E-5
ACO1	aconitase 1, soluble	-0.567	1.72E-5
CLYBL	citrate lyase beta like	-0.567	1.74E-5
SFMBT1	Scm-like with four mbt domains 1	-0.566	1.76E-5
MEX3C	mex-3 homolog C (C. elegans)	-0.566	1.76E-5
LY9	lymphocyte antigen 9	-0.566	1.76E-5
ZNF577	zinc finger protein 577	-0.566	1.76E-5
CCR7	chemokine (C-C motif) receptor 7	-0.566	1.77E-5
FIGNL1	fidgetin-like 1	-0.565	1.77E-5
BRD8	bromodomain containing 8	-0.565	1.77E-5

Gene symbol	Gene title	ρ	Adjusted P
TTC5	tetratricopeptide repeat domain 5	-0.564	1.85E-5
DSC1	desmocollin 1	-0.564	1.85E-5
MDN1	MDN1, midasin homolog (yeast)	-0.564	1.85E-5
IKZF3	IKAROS family zinc finger 3 (Aiolos)	-0.564	1.85E-5
S1PR1	sphingosine-1-phosphate receptor 1	-0.564	1.85E-5
SEL1L3	sel-1 suppressor of lin-12-like 3 (C. elegans)	-0.563	1.85E-5
NAT10	N-acetyltransferase 10 (GCN5-related)	-0.563	1.85E-5
ZNF383	zinc finger protein 383	-0.563	1.86E-5
ARSK	arylsulfatase family, member K	-0.562	1.92E-5
WDR89	WD repeat domain 89	-0.562	1.92E-5
PRKCH	protein kinase C, eta	-0.562	1.97E-5
CREBZF	CREB/ATF bZIP transcription factor	-0.561	1.97E-5
ASTE1	asteroid homolog 1 (Drosophila)	-0.561	1.97E-5
HIVEP2	human immunodeficiency virus type I enhancer binding protein 2	-0.561	1.97E-5
CSTF1	cleavage stimulation factor, 3' pre-RNA, subunit 1, 50kDa	-0.561	1.97E-5
TRAF5	TNF receptor-associated factor 5	-0.561	1.97E-5
NUMA1	nuclear mitotic apparatus protein 1	-0.561	1.98E-5
BMPR1A	bone morphogenetic protein receptor, type IA	-0.560	2.01E-5
LIMA1	LIM domain and actin binding 1	-0.560	2.02E-5
HDHD2	haloacid dehalogenase-like hydrolase domain containing 2	-0.560	2.03E-5
LCMT2	leucine carboxyl methyltransferase 2	-0.559	2.06E-5
CCDC76	coiled-coil domain containing 76	-0.559	2.09E-5
SHPRH	SNF2 histone linker PHD RING helicase	-0.559	2.09E-5
ZC3H6	zinc finger CCCH-type containing 6	-0.559	2.09E-5
TMEM117	transmembrane protein 117	-0.559	2.10E-5
LRCH3	leucine-rich repeats and calponin homology (CH) domain containing 3	-0.558	2.19E-5
CHD6	chromodomain helicase DNA binding protein 6	-0.557	2.23E-5
ZNF566	zinc finger protein 566	-0.556	2.32E-5
ZNF623	zinc finger protein 623	-0.556	2.33E-5
FNTA	farnesyltransferase, CAAX box, alpha	-0.555	2.43E-5
PEX12	peroxisomal biogenesis factor 12	-0.555	2.46E-5
ZMYND11	zinc finger, MYND domain containing 11	-0.555	2.46E-5
MTR	5-methyltetrahydrofolate-homocysteine methyltransferase	-0.555	2.46E-5
DNAJC19	DnaJ (Hsp40) homolog, subfamily C, member 19	-0.554	2.47E-5
NFATC3	nuclear factor of activated T-cells, cytoplasmic, calcineurin-dependent 3	-0.554	2.47E-5
PPP2R2D	protein phosphatase 2, regulatory subunit B, delta	-0.554	2.47E-5
RPS25	ribosomal protein S25	-0.554	2.48E-5
MLL	myeloid/lymphoid or mixed-lineage leukemia (trithorax homolog, Drosophila)	-0.554	2.49E-5
C14orf1	chromosome 14 open reading frame 1	-0.554	2.49E-5
IKBKB	inhibitor of kappa light polypeptide gene enhancer in B-cells, kinase beta	-0.553	2.53E-5
ZNF461	zinc finger protein 461	-0.553	2.54E-5
PLCG1	phospholipase C, gamma 1	-0.552	2.59E-5
ZNF174	zinc finger protein 174	-0.552	2.67E-5
ZNF226	zinc finger protein 226	-0.552	2.68E-5

Gene symbol	Gene title	ρ	Adjusted P
TCP11L1	t-complex 11 (mouse)-like 1	-0.551	2.70E-5
POLR3A	polymerase (RNA) III (DNA directed) polypeptide A, 155kDa	-0.550	2.77E-5
FTSJD1	FtsJ methyltransferase domain containing 1	-0.550	2.77E-5
RRP8	ribosomal RNA processing 8, methyltransferase, homolog (yeast)	-0.550	2.77E-5
SNX25	sorting nexin 25	-0.550	2.77E-5
PPP3CC	protein phosphatase 3, catalytic subunit, gamma isozyme	-0.550	2.77E-5
CCDC141	coiled-coil domain containing 141	-0.550	2.77E-5
TRMT11	tRNA methyltransferase 11 homolog (S. cerevisiae)	-0.550	2.77E-5
BBS10	Bardet-Biedl syndrome 10	-0.550	2.80E-5
UTP20	UTP20, small subunit (SSU) processome component, homolog (yeast)	-0.549	2.85E-5
SIRT1	sirtuin 1	-0.549	2.85E-5
KDELC2	KDEL (Lys-Asp-Glu-Leu) containing 2	-0.549	2.85E-5
ZNF483	zinc finger protein 483	-0.549	2.85E-5
TCF20	transcription factor 20 (AR1)	-0.549	2.85E-5
DNAJA3	DnaJ (Hsp40) homolog, subfamily A, member 3	-0.548	2.90E-5
CARD11	caspase recruitment domain family, member 11	-0.547	3.02E-5
ZNF17	zinc finger protein 17	-0.547	3.04E-5
PITPNC1	phosphatidylinositol transfer protein, cytoplasmic 1	-0.547	3.04E-5
HECTD1	HECT domain containing 1	-0.547	3.04E-5
DDHD2	DDHD domain containing 2	-0.546	3.14E-5
CTCF	CCCTC-binding factor (zinc finger protein)	-0.546	3.16E-5
TSC1	tuberous sclerosis 1	-0.546	3.16E-5
CASP8AP2	caspase 8 associated protein 2	-0.546	3.16E-5
KIAA0748	KIAA0748	-0.545	3.16E-5
ACACB	acetyl-CoA carboxylase beta	-0.545	3.16E-5
C12orf65	chromosome 12 open reading frame 65	-0.545	3.16E-5
KPNA5	karyopherin alpha 5 (importin alpha 6)	-0.545	3.18E-5
DTWD2	DTW domain containing 2	-0.545	3.24E-5
TRIB2	tribbles homolog 2 (Drosophila)	-0.544	3.27E-5
WDR53	WD repeat domain 53	-0.544	3.27E-5
C10orf137	chromosome 10 open reading frame 137	-0.544	3.27E-5
ASXL1	additional sex combs like 1 (Drosophila)	-0.544	3.27E-5
ZNF544	zinc finger protein 544	-0.544	3.27E-5
ZNF480	zinc finger protein 480	-0.544	3.28E-5
ELK4	ELK4, ETS-domain protein (SRF accessory protein 1)	-0.544	3.28E-5
HERC4	hect domain and RLD 4	-0.544	3.28E-5
ZNF441	zinc finger protein 441	-0.544	3.28E-5
ZNF337	zinc finger protein 337	-0.543	3.34E-5
SLAMF6	SLAM family member 6	-0.542	3.45E-5
ZNF141	zinc finger protein 141	-0.542	3.45E-5
FNBP4	formin binding protein 4	-0.542	3.47E-5
CD84	CD84 molecule	-0.542	3.52E-5
ZNF600	zinc finger protein 600	-0.541	3.55E-5
ATP10A	ATPase, class V, type 10A	-0.541	3.58E-5
NOC3L	nucleolar complex associated 3 homolog (S. cerevisiae)	-0.541	3.58E-5
CYP4V2	cytochrome P450, family 4, subfamily V, polypeptide 2	-0.541	3.60E-5
ELP2	elongation protein 2 homolog (S. cerevisiae)	-0.541	3.63E-5

Gene symbol	Gene title	ρ	Adjusted P
GPAM	glycerol-3-phosphate acyltransferase, mitochondrial	-0.540	3.64E-5
LEF1	lymphoid enhancer-binding factor 1	-0.540	3.64E-5
PPIP5K1	diphosphoinositol pentakisphosphate kinase 1	-0.540	3.64E-5
ABCC5	ATP-binding cassette, sub-family C (CFTR/MRP), member 5	-0.540	3.64E-5
SPTAN1	spectrin, alpha, non-erythrocytic 1 (alpha-fodrin)	-0.540	3.64E-5
GIMAP7	GTPase, IMAP family member 7	-0.540	3.64E-5
PIGU	phosphatidylinositol glycan anchor biosynthesis, class U	-0.540	3.64E-5
ZNF329	zinc finger protein 329	-0.540	3.64E-5
PCNXL2	pecanex-like 2 (Drosophila)	-0.540	3.64E-5
PEX1	peroxisomal biogenesis factor 1	-0.540	3.67E-5
SP4	Sp4 transcription factor	-0.539	3.69E-5
EMG1	EMG1 nucleolar protein homolog (S. cerevisiae)	-0.539	3.72E-5
PIK3R4	phosphoinositide-3-kinase, regulatory subunit 4	-0.539	3.73E-5
PCYOX1	prenylcysteine oxidase 1	-0.538	3.87E-5
RQCD1	RCD1 required for cell differentiation1 homolog (S. pombe)	-0.538	3.87E-5
PPP1R13B	protein phosphatase 1, regulatory (inhibitor) subunit 13B	-0.538	3.89E-5
ZNF12	zinc finger protein 12	-0.538	3.89E-5
AP4B1	adaptor-related protein complex 4, beta 1 subunit	-0.538	3.89E-5
KIAA1430	KIAA1430	-0.538	3.89E-5
MRS2	MRS2 magnesium homeostasis factor homolog (S. cerevisiae)	-0.538	3.90E-5
SMARCC1	SWI/SNF related, matrix associated, actin dependent regulator of chromatin, subfamily c, member 1	-0.537	3.97E-5
ELP3	elongation protein 3 homolog (S. cerevisiae)	-0.537	3.97E-5
KIAA0562	KIAA0562	-0.537	3.97E-5
KLF12	Kruppel-like factor 12	-0.537	4.00E-5
ABLIM1	actin binding LIM protein 1	-0.536	4.03E-5
ZNF83	zinc finger protein 83	-0.536	4.06E-5
TAS2R3	taste receptor, type 2, member 3	-0.536	4.12E-5
DGCR8	DiGeorge syndrome critical region gene 8	-0.536	4.13E-5
ABCD4	ATP-binding cassette, sub-family D (ALD), member 4	-0.536	4.13E-5
NUP35	nucleoporin 35kDa	-0.535	4.15E-5
CUBN	cubilin (intrinsic factor-cobalamin receptor)	-0.535	4.16E-5
ZFP82	zinc finger protein 82 homolog (mouse)	-0.535	4.16E-5
FAM159A	family with sequence similarity 159, member A	-0.535	4.17E-5
CAPRIN2	caprin family member 2	-0.535	4.17E-5
SIN3A	SIN3 homolog A, transcription regulator (yeast)	-0.535	4.17E-5
IKZF1	IKAROS family zinc finger 1 (Ikaros)	-0.535	4.17E-5
EPHX2	epoxide hydrolase 2, cytoplasmic	-0.535	4.17E-5
ZNF234	zinc finger protein 234	-0.535	4.19E-5
MED23	mediator complex subunit 23	-0.535	4.19E-5
TRIM52	tripartite motif-containing 52	-0.534	4.22E-5
INPP4B	inositol polyphosphate-4-phosphatase, type II, 105kDa	-0.534	4.30E-5
POLH	polymerase (DNA directed), eta	-0.534	4.30E-5
KIAA1826	KIAA1826	-0.534	4.30E-5
FAM134C	family with sequence similarity 134, member C	-0.534	4.30E-5
HARS2	histidyl-tRNA synthetase 2, mitochondrial (putative)	-0.534	4.30E-5
HPS3	Hermansky-Pudlak syndrome 3	-0.534	4.30E-5
RASGRP1	RAS guanyl releasing protein 1 (calcium and DAG-regulated)	-0.533	4.30E-5
PTCD2	pentatricopeptide repeat domain 2	-0.533	4.30E-5

Gene symbol	Gene title	ρ	Adjusted *P*
DGKA	diacylglycerol kinase, alpha 80kDa	-0.533	4.31E-5
ZNF3	zinc finger protein 3	-0.533	4.31E-5
SERGEF	secretion regulating guanine nucleotide exchange factor	-0.533	4.31E-5
MTERFD2	MTERF domain containing 2	-0.533	4.31E-5
HMG20A	high-mobility group 20A	-0.533	4.35E-5
SNRNP70	small nuclear ribonucleoprotein 70kDa (U1)	-0.533	4.38E-5
MRPS14	mitochondrial ribosomal protein S14	-0.532	4.45E-5
TGIF2	TGFB-induced factor homeobox 2	-0.532	4.45E-5
ITK	IL2-inducible T-cell kinase	-0.532	4.50E-5
SMARCC2	SWI/SNF related, matrix associated, actin dependent regulator of chromatin, subfamily c, member 2	-0.531	4.51E-5
TUG1	taurine upregulated 1 (non-protein coding)	-0.531	4.51E-5
NDRG2	NDRG family member 2	-0.531	4.51E-5
HLCS	holocarboxylase synthetase (biotin-(proprionyl-CoA-carboxylase (ATP-hydrolysing)) ligase)	-0.531	4.51E-5
C6orf130	chromosome 6 open reading frame 130	-0.531	4.51E-5
ITPR1	inositol 1,4,5-triphosphate receptor, type 1	-0.531	4.51E-5
BRWD1	bromodomain and WD repeat domain containing 1	-0.531	4.51E-5
ZNF14	zinc finger protein 14	-0.531	4.51E-5
RFXAP	regulatory factor X-associated protein	-0.531	4.51E-5
GCNT4	glucosaminyl (N-acetyl) transferase 4, core 2	-0.531	4.51E-5
MAP4K1	mitogen-activated protein kinase kinase kinase kinase 1	-0.531	4.51E-5
LCMT1	leucine carboxyl methyltransferase 1	-0.531	4.55E-5
TMEM161B	transmembrane protein 161B	-0.530	4.62E-5
CYFIP2	cytoplasmic FMR1 interacting protein 2	-0.530	4.64E-5
C3orf17	chromosome 3 open reading frame 17	-0.530	4.64E-5
ZNF616	zinc finger protein 616	-0.530	4.66E-5
MIPEP	mitochondrial intermediate peptidase	-0.529	4.80E-5
LPIN1	lipin 1	-0.529	4.80E-5
C22orf30	chromosome 22 open reading frame 30	-0.529	4.80E-5
INPP4A	inositol polyphosphate-4-phosphatase, type I, 107kDa	-0.529	4.89E-5
UBASH3A	ubiquitin associated and SH3 domain containing A	-0.528	5.01E-5
KLHL9	kelch-like 9 (Drosophila)	-0.528	5.01E-5
LANCL1	LanC lantibiotic synthetase component C-like 1 (bacterial)	-0.528	5.01E-5
RTTN	rotatin	-0.528	5.03E-5
VPS13D	vacuolar protein sorting 13 homolog D (S. cerevisiae)	-0.528	5.07E-5
TRAF1	TNF receptor-associated factor 1	-0.527	5.14E-5
RNF34	ring finger protein 34	-0.527	5.14E-5
C17orf68	chromosome 17 open reading frame 68	-0.527	5.14E-5
LDB1	LIM domain binding 1	-0.527	5.15E-5
ZNF460	zinc finger protein 460	-0.527	5.15E-5
ALDH9A1	aldehyde dehydrogenase 9 family, member A1	-0.527	5.15E-5
ADNP	activity-dependent neuroprotector homeobox	-0.527	5.15E-5
SNRNP200	small nuclear ribonucleoprotein 200kDa (U5)	-0.527	5.15E-5
DCAF17	DDB1 and CUL4 associated factor 17	-0.526	5.19E-5
SPATA5	spermatogenesis associated 5	-0.526	5.19E-5
PARN	poly(A)-specific ribonuclease (deadenylation nuclease)	-0.526	5.19E-5
MLL5	myeloid/lymphoid or mixed-lineage leukemia 5 (trithorax homolog, Drosophila)	-0.526	5.19E-5

Gene symbol	Gene title	ρ	Adjusted *P*
NARS2	asparaginyl-tRNA synthetase 2, mitochondrial (putative)	-0.526	5.19E-5
POC5	POC5 centriolar protein homolog (Chlamydomonas)	-0.526	5.20E-5
FAIM3	Fas apoptotic inhibitory molecule 3	-0.526	5.20E-5
TRAPPC10	trafficking protein particle complex 10	-0.526	5.20E-5
CTAGE5	CTAGE family, member 5	-0.526	5.22E-5
METTL6	methyltransferase like 6	-0.526	5.27E-5
STK39	serine threonine kinase 39	-0.525	5.31E-5
GTF2H1	general transcription factor IIH, polypeptide 1, 62kDa	-0.525	5.35E-5
BAT2L1	HLA-B associated transcript 2-like 1	-0.525	5.44E-5
SLC46A3	solute carrier family 46, member 3	-0.525	5.44E-5
TMEM68	transmembrane protein 68	-0.525	5.47E-5
LCLAT1	lysocardiolipin acyltransferase 1	-0.525	5.47E-5
USP13	ubiquitin specific peptidase 13 (isopeptidase T-3)	-0.524	5.51E-5
PYHIN1	pyrin and HIN domain family, member 1	-0.524	5.60E-5
FCRL3	Fc receptor-like 3	-0.524	5.63E-5
HEATR1	HEAT repeat containing 1	-0.524	5.63E-5
MPP6	membrane protein, palmitoylated 6 (MAGUK p55 subfamily member 6)	-0.523	5.74E-5
BACH2	BTB and CNC homology 1, basic leucine zipper transcription factor 2	-0.523	5.76E-5
MKRN2	makorin ring finger protein 2	-0.523	5.76E-5
PCID2	PCI domain containing 2	-0.523	5.79E-5
ZNF101	zinc finger protein 101	-0.523	5.79E-5
TMTC3	transmembrane and tetratricopeptide repeat containing 3	-0.523	5.80E-5
IKBKAP	inhibitor of kappa light polypeptide gene enhancer in B-cells, kinase complex-associated protein	-0.523	5.80E-5
C11orf61	chromosome 11 open reading frame 61	-0.523	5.83E-5
ZCCHC7	zinc finger, CCHC domain containing 7	-0.522	5.86E-5
SLC38A1	solute carrier family 38, member 1	-0.522	5.86E-5
ZFP161	zinc finger protein 161 homolog (mouse)	-0.522	5.91E-5
STRBP	spermatid perinuclear RNA binding protein	-0.522	5.92E-5
SKAP1	src kinase associated phosphoprotein 1	-0.522	5.92E-5
C17orf48	chromosome 17 open reading frame 48	-0.522	5.92E-5
ZNF26	zinc finger protein 26	-0.522	5.94E-5
PPP3CB	protein phosphatase 3, catalytic subunit, beta isozyme	-0.522	5.94E-5
ZNF43	zinc finger protein 43	-0.521	5.96E-5
CHMP7	CHMP family, member 7	-0.521	5.96E-5
FBXO21	F-box protein 21	-0.521	5.96E-5
MYO6	myosin VI	-0.521	6.04E-5
UQCC	ubiquinol-cytochrome c reductase complex chaperone	-0.521	6.04E-5
NUP153	nucleoporin 153kDa	-0.521	6.07E-5
ENOSF1	enolase superfamily member 1	-0.521	6.07E-5
PARP15	poly (ADP-ribose) polymerase family, member 15	-0.521	6.07E-5
NUBPL	nucleotide binding protein-like	-0.521	6.07E-5
ALDH18A1	aldehyde dehydrogenase 18 family, member A1	-0.521	6.07E-5
MED17	mediator complex subunit 17	-0.520	6.12E-5
APPBP2	amyloid beta precursor protein (cytoplasmic tail) binding protein 2	-0.520	6.14E-5
EVL	Enah/Vasp-like	-0.520	6.14E-5

Gene symbol	Gene title	ρ	Adjusted *P*
GOT1	glutamic-oxaloacetic transaminase 1, soluble (aspartate aminotransferase 1)	-0.520	6.14E-5
WDR59	WD repeat domain 59	-0.520	6.18E-5
RFWD3	ring finger and WD repeat domain 3	-0.520	6.18E-5
PHF15	PHD finger protein 15	-0.520	6.21E-5
GRAMD3	GRAM domain containing 3	-0.520	6.21E-5
ZBED5	zinc finger, BED-type containing 5	-0.520	6.23E-5
IMPDH2	IMP (inosine 5'-monophosphate) dehydrogenase 2	-0.520	6.23E-5
NOL9	nucleolar protein 9	-0.520	6.23E-5
GFM1	G elongation factor, mitochondrial 1	-0.519	6.25E-5
RPL15	ribosomal protein L15	-0.519	6.25E-5
TADA2A	transcriptional adaptor 2A	-0.519	6.25E-5
MIER3	mesoderm induction early response 1, family member 3	-0.519	6.27E-5
ACTR5	ARP5 actin-related protein 5 homolog (yeast)	-0.519	6.27E-5
PPM1K	protein phosphatase, Mg2+/Mn2+ dependent, 1K	-0.519	6.27E-5
NUP88	nucleoporin 88kDa	-0.519	6.30E-5
NUP133	nucleoporin 133kDa	-0.519	6.30E-5
TUBGCP5	tubulin, gamma complex associated protein 5	-0.519	6.36E-5
SF3B3	splicing factor 3b, subunit 3, 130kDa	-0.518	6.45E-5
COQ6	coenzyme Q6 homolog, monooxygenase (S. cerevisiae)	-0.518	6.45E-5
ZNF420	zinc finger protein 420	-0.518	6.45E-5
BAZ1B	bromodomain adjacent to zinc finger domain, 1B	-0.518	6.45E-5
MCM8	minichromosome maintenance complex component 8	-0.518	6.45E-5
SPATA13	spermatogenesis associated 13	-0.518	6.45E-5
C11orf30	chromosome 11 open reading frame 30	-0.518	6.47E-5
SLC5A3	solute carrier family 5 (sodium/myo-inositol cotransporter), member 3	-0.518	6.47E-5
MCM3AP	minichromosome maintenance complex component 3 associated protein	-0.518	6.47E-5
TARSL2	threonyl-tRNA synthetase-like 2	-0.518	6.48E-5
RSPRY1	ring finger and SPRY domain containing 1	-0.518	6.48E-5
USP28	ubiquitin specific peptidase 28	-0.518	6.48E-5
TCTN3	tectonic family member 3	-0.518	6.48E-5
KIAA0586	KIAA0586	-0.518	6.49E-5
ZNF507	zinc finger protein 507	-0.518	6.49E-5
PDCD11	programmed cell death 11	-0.518	6.49E-5
TDRKH	tudor and KH domain containing	-0.517	6.54E-5
RASGRF2	Ras protein-specific guanine nucleotide-releasing factor 2	-0.517	6.57E-5
KIF21A	kinesin family member 21A	-0.517	6.57E-5
CEP164	centrosomal protein 164kDa	-0.517	6.62E-5
MYO9A	myosin IXA	-0.516	6.75E-5
ZNF493	zinc finger protein 493	-0.516	6.79E-5
HDDC2	HD domain containing 2	-0.516	6.89E-5
RPRD2	regulation of nuclear pre-mRNA domain containing 2	-0.516	6.89E-5
AGL	amylo-alpha-1, 6-glucosidase, 4-alpha-glucanotransferase	-0.516	6.89E-5
GALT	galactose-1-phosphate uridylyltransferase	-0.516	6.93E-5
FHIT	fragile histidine triad gene	-0.516	6.93E-5
NFS1	NFS1 nitrogen fixation 1 homolog (S. cerevisiae)	-0.515	6.98E-5
ADAL	adenosine deaminase-like	-0.515	7.04E-5

Gene symbol	Gene title	ρ	Adjusted *P*
ZNF571	zinc finger protein 571	-0.515	7.16E-5
NPCDR1	nasopharyngeal carcinoma, down-regulated 1	-0.515	7.16E-5
XPNPEP3	X-prolyl aminopeptidase (aminopeptidase P) 3, putative	-0.515	7.20E-5
CYTH2	cytohesin 2	-0.515	7.20E-5
N6AMT1	N-6 adenine-specific DNA methyltransferase 1 (putative)	-0.514	7.24E-5
SBNO1	strawberry notch homolog 1 (Drosophila)	-0.514	7.24E-5
COG4	component of oligomeric golgi complex 4	-0.514	7.31E-5
ZNF567	zinc finger protein 567	-0.514	7.37E-5
MINA	MYC induced nuclear antigen	-0.514	7.37E-5
POLR1E	polymerase (RNA) I polypeptide E, 53kDa	-0.514	7.37E-5
TRIM37	tripartite motif-containing 37	-0.514	7.37E-5
DIDO1	death inducer-obliterator 1	-0.514	7.37E-5
PEX5	peroxisomal biogenesis factor 5	-0.514	7.37E-5
ZNF718	zinc finger protein 718	-0.514	7.37E-5
C20orf177	chromosome 20 open reading frame 177	-0.514	7.37E-5
NFX1	nuclear transcription factor, X-box binding 1	-0.513	7.44E-5
ANK3	ankyrin 3, node of Ranvier (ankyrin G)	-0.513	7.48E-5
NR2C2	nuclear receptor subfamily 2, group C, member 2	-0.513	7.53E-5
FAM188A	family with sequence similarity 188, member A	-0.513	7.53E-5
INPP5B	inositol polyphosphate-5-phosphatase, 75kDa	-0.512	7.71E-5
KIAA1147	KIAA1147	-0.512	7.71E-5
OXCT1	3-oxoacid CoA transferase 1	-0.512	7.71E-5
C6orf106	chromosome 6 open reading frame 106	-0.512	7.71E-5
RBL1	retinoblastoma-like 1 (p107)	-0.512	7.76E-5
TRERF1	transcriptional regulating factor 1	-0.512	7.76E-5
RAB22A	RAB22A, member RAS oncogene family	-0.512	7.82E-5
MBTPS1	membrane-bound transcription factor peptidase, site 1	-0.512	7.89E-5
PREPL	prolyl endopeptidase-like	-0.511	7.89E-5
MLH3	mutL homolog 3 (E. coli)	-0.511	7.89E-5
MAP3K12	mitogen-activated protein kinase kinase kinase 12	-0.511	7.90E-5
NEK1	NIMA (never in mitosis gene a)-related kinase 1	-0.511	7.90E-5
TCTN2	tectonic family member 2	-0.511	7.92E-5
MORC2	MORC family CW-type zinc finger 2	-0.511	7.92E-5
ANAPC1	anaphase promoting complex subunit 1	-0.511	7.97E-5
TGFBR1	transforming growth factor, beta receptor 1	-0.511	7.97E-5
C6orf170	chromosome 6 open reading frame 170	-0.511	8.02E-5
ZZZ3	zinc finger, ZZ-type containing 3	-0.511	8.02E-5
ASB7	ankyrin repeat and SOCS box-containing 7	-0.510	8.07E-5
RALGAPB	Ral GTPase activating protein, beta subunit (non-catalytic)	-0.510	8.07E-5
SMURF2	SMAD specific E3 ubiquitin protein ligase 2	-0.510	8.14E-5
FBXO28	F-box protein 28	-0.510	8.18E-5
TCF7	transcription factor 7 (T-cell specific, HMG-box)	-0.510	8.18E-5
ITGA6	integrin, alpha 6	-0.510	8.18E-5
WDR75	WD repeat domain 75	-0.510	8.25E-5
SAAL1	serum amyloid A-like 1	-0.509	8.41E-5
ANXA6	annexin A6	-0.509	8.43E-5
MRPL39	mitochondrial ribosomal protein L39	-0.509	8.43E-5
KLHL3	kelch-like 3 (Drosophila)	-0.509	8.43E-5
SEMA4C	sema domain, immunoglobulin domain (Ig), transmembrane	-0.509	8.43E-5

Gene symbol	Gene title	ρ	Adjusted *P*
	domain (TM) and short cytoplasmic domain, (semaphorin) 4C		
INPP5F	inositol polyphosphate-5-phosphatase F	-0.509	8.48E-5
DDX31	DEAD (Asp-Glu-Ala-Asp) box polypeptide 31	-0.509	8.48E-5
GABPB2	GA binding protein transcription factor, beta subunit 2	-0.509	8.50E-5
UMPS	uridine monophosphate synthetase	-0.509	8.50E-5
PRKAB1	protein kinase, AMP-activated, beta 1 non-catalytic subunit	-0.509	8.50E-5
LRIG2	leucine-rich repeats and immunoglobulin-like domains 2	-0.509	8.50E-5
ZNF160	zinc finger protein 160	-0.509	8.56E-5
BCKDHB	branched chain keto acid dehydrogenase E1, beta polypeptide	-0.508	8.63E-5
LINS	lines homolog (Drosophila)	-0.508	8.67E-5
HELB	helicase (DNA) B	-0.508	8.67E-5
MFAP1	microfibrillar-associated protein 1	-0.508	8.67E-5
ERCC6L2	Excision Repair Cross-Complementation Group 6-Like 2	-0.508	8.92E-5
CYP20A1	cytochrome P450, family 20, subfamily A, polypeptide 1	-0.507	8.99E-5
CDC37L1	cell division cycle 37 homolog (S. cerevisiae)-like 1	-0.507	9.00E-5
SUV420H1	suppressor of variegation 4-20 homolog 1 (Drosophila)	-0.507	9.00E-5
DSTNP2	destrin (actin depolymerizing factor) pseudogene 2	-0.507	9.00E-5
TRMT5	TRM5 tRNA methyltransferase 5 homolog (S. cerevisiae)	-0.507	9.00E-5
ZMYM1	zinc finger, MYM-type 1	-0.507	9.00E-5
DIS3L	DIS3 mitotic control homolog (S. cerevisiae)-like	-0.507	9.00E-5
FANCM	Fanconi anemia, complementation group M	-0.507	9.03E-5
DEPDC5	DEP domain containing 5	-0.507	9.03E-5
EXOSC3	exosome component 3	-0.507	9.03E-5
CWF19L2	CWF19-like 2, cell cycle control (S. pombe)	-0.506	9.09E-5
EFCAB4B	EF-hand calcium binding domain 4B	-0.506	9.09E-5
ZNF295	zinc finger protein 295	-0.506	9.13E-5
ZNF345	zinc finger protein 345	-0.506	9.13E-5
AKT3	v-akt murine thymoma viral oncogene homolog 3 (protein kinase B, gamma)	-0.506	9.14E-5
CRY1	cryptochrome 1 (photolyase-like)	-0.506	9.14E-5
ZFP90	zinc finger protein 90 homolog (mouse)	-0.506	9.14E-5
CEP250	centrosomal protein 250kDa	-0.506	9.14E-5
PRPF38B	PRP38 pre-mRNA processing factor 38 (yeast) domain containing B	-0.506	9.17E-5
VTI1A	vesicle transport through interaction with t-SNAREs homolog 1A (yeast)	-0.506	9.17E-5
GIN1	gypsy retrotransposon integrase 1	-0.506	9.17E-5
FOXJ3	forkhead box J3	-0.505	9.31E-5
METTL4	methyltransferase like 4	-0.505	9.33E-5
WDR35	WD repeat domain 35	-0.505	9.33E-5
TRDMT1	tRNA aspartic acid methyltransferase 1	-0.505	9.33E-5
RNF219	ring finger protein 219	-0.505	9.45E-5
EFHA2	EF-hand domain family, member A2	-0.505	9.48E-5
DENND1C	DENN/MADD domain containing 1C	-0.504	9.64E-5
BDP1	B double prime 1, subunit of RNA polymerase III transcription initiation factor IIIB	-0.504	9.64E-5
HKR1	HKR1, GLI-Kruppel zinc finger family member	-0.504	9.69E-5
NEK9	NIMA (never in mitosis gene a)- related kinase 9	-0.504	9.69E-5
ZNF187	zinc finger protein 187	-0.504	9.69E-5

Gene symbol	Gene title	*ρ*	Adjusted *P*
ZNF18	zinc finger protein 18	-0.504	9.78E-5
EPC1	enhancer of polycomb homolog 1 (Drosophila)	-0.504	9.79E-5
FAM113B	family with sequence similarity 113, member B	-0.504	9.79E-5
ZNF573	zinc finger protein 573	-0.504	9.79E-5
SPICE1	spindle and centriole associated protein 1	-0.504	9.79E-5
ZBTB41	zinc finger and BTB domain containing 41	-0.504	9.79E-5
MEAF6	MYST/Esa1-associated factor 6	-0.504	9.87E-5
MTMR4	myotubularin related protein 4	-0.503	9.96E-5
ZNF180	zinc finger protein 180	-0.503	1.00E-4
IMP3	IMP3, U3 small nucleolar ribonucleoprotein, homolog (yeast)	-0.503	1.01E-4
URI1	URI1, Prefoldin-Like Chaperone	-0.503	1.01E-4
MRPS7	mitochondrial ribosomal protein S7	-0.502	1.03E-4
SC5DL	sterol-C5-desaturase (ERG3 delta-5-desaturase homolog, S. cerevisiae)-like	-0.502	1.03E-4
CDC5L	CDC5 cell division cycle 5-like (S. pombe)	-0.502	1.03E-4
ABCD2	ATP-binding cassette, sub-family D (ALD), member 2	-0.502	1.04E-4
NOP2	NOP2 nucleolar protein homolog (yeast)	-0.502	1.04E-4
ZBTB40	zinc finger and BTB domain containing 40	-0.502	1.04E-4
MAP3K7	mitogen-activated protein kinase kinase kinase 7	-0.502	1.05E-4
WHSC1L1	Wolf-Hirschhorn syndrome candidate 1-like 1	-0.502	1.05E-4
DGKE	diacylglycerol kinase, epsilon 64kDa	-0.502	1.05E-4
RNF214	ring finger protein 214	-0.501	1.08E-4
GON4L	gon-4-like (C. elegans)	-0.501	1.08E-4
TSGA10	testis specific, 10	-0.501	1.08E-4
USP54	ubiquitin specific peptidase 54	-0.501	1.08E-4
PTPLB	protein tyrosine phosphatase-like (proline instead of catalytic arginine), member b	-0.501	1.09E-4
POLG2	polymerase (DNA directed), gamma 2, accessory subunit	-0.500	1.10E-4
ZNF528	zinc finger protein 528	-0.500	1.10E-4
C4orf43	chromosome 4 open reading frame 43	-0.500	1.10E-4
ZNF202	zinc finger protein 202	-0.500	1.10E-4
MRE11A	MRE11 meiotic recombination 11 homolog A (S. cerevisiae)	-0.500	1.10E-4
RBBP5	retinoblastoma binding protein 5	-0.500	1.10E-4
STAT4	signal transducer and activator of transcription 4	-0.500	1.11E-4
ZC3H14	zinc finger CCCH-type containing 14	-0.500	1.11E-4
MSH2	mutS homolog 2, colon cancer, nonpolyposis type 1 (E. coli)	-0.500	1.12E-4
ORC2	origin recognition complex, subunit 2	-0.500	1.12E-4
MRPL30	mitochondrial ribosomal protein L30	-0.500	1.12E-4
NIPAL3	NIPA-like domain containing 3	-0.500	1.12E-4
LNPEP	leucyl/cystinyl aminopeptidase	-0.500	1.12E-4
TGS1	trimethylguanosine synthase 1	-0.500	1.12E-4
CEP192	centrosomal protein 192kDa	-0.499	1.12E-4
CTR9	Ctr9, Paf1/RNA polymerase II complex component, homolog (S. cerevisiae)	-0.499	1.12E-4
SFXN2	sideroflexin 2	-0.499	1.12E-4
TCEA3	transcription elongation factor A (SII), 3	-0.499	1.12E-4
CAPN7	calpain 7	-0.499	1.13E-4
SMAD4	SMAD family member 4	-0.499	1.13E-4
LSG1	large subunit GTPase 1 homolog (S. cerevisiae)	-0.499	1.14E-4

Gene symbol	Gene title	ρ	Adjusted P
GOLGA3	golgin A3	-0.499	1.14E-4
CCDC99	coiled-coil domain containing 99	-0.499	1.14E-4
ACAD10	acyl-CoA dehydrogenase family, member 10	-0.499	1.14E-4
RAD17	RAD17 homolog (S. pombe)	-0.499	1.15E-4
YEATS4	YEATS domain containing 4	-0.498	1.16E-4
HIBADH	3-hydroxyisobutyrate dehydrogenase	-0.498	1.16E-4
GRLF1	glucocorticoid receptor DNA binding factor 1	-0.498	1.16E-4
BNIP3	BCL2/adenovirus E1B 19kDa interacting protein 3	-0.498	1.18E-4
SGCB	sarcoglycan, beta (43kDa dystrophin-associated glycoprotein)	-0.498	1.18E-4
WDR3	WD repeat domain 3	-0.498	1.19E-4
MAPK9	mitogen-activated protein kinase 9	-0.498	1.20E-4
KDM4C	lysine (K)-specific demethylase 4C	-0.497	1.20E-4
C6orf204	chromosome 6 open reading frame 204	-0.497	1.20E-4
IWS1	IWS1 homolog (S. cerevisiae)	-0.497	1.20E-4
TERF2	telomeric repeat binding factor 2	-0.497	1.20E-4
TBC1D19	TBC1 domain family, member 19	-0.497	1.21E-4
JRKL	jerky homolog-like (mouse)	-0.497	1.22E-4
USPL1	ubiquitin specific peptidase like 1	-0.497	1.22E-4
ZNF426	zinc finger protein 426	-0.497	1.22E-4
ANKRD26	ankyrin repeat domain 26	-0.497	1.22E-4
SLC23A2	solute carrier family 23 (nucleobase transporters), member 2	-0.497	1.22E-4
AUH	AU RNA binding protein/enoyl-CoA hydratase	-0.497	1.22E-4
RAD18	RAD18 homolog (S. cerevisiae)	-0.497	1.23E-4
SLFN5	schlafen family member 5	-0.496	1.24E-4
TRAF3IP3	TRAF3 interacting protein 3	-0.496	1.24E-4
SLC4A7	solute carrier family 4, sodium bicarbonate cotransporter, member 7	-0.496	1.24E-4
DCUN1D4	DCN1, defective in cullin neddylation 1, domain containing 4 (S. cerevisiae)	-0.496	1.24E-4
ATG2B	ATG2 autophagy related 2 homolog B (S. cerevisiae)	-0.496	1.24E-4
PRMT7	protein arginine methyltransferase 7	-0.496	1.24E-4
FARS2	phenylalanyl-tRNA synthetase 2, mitochondrial	-0.496	1.24E-4
OXNAD1	oxidoreductase NAD-binding domain containing 1	-0.496	1.24E-4
CD3D	CD3d molecule, delta (CD3-TCR complex)	-0.496	1.25E-4
DROSHA	drosha, ribonuclease type III	-0.496	1.25E-4
ZNF680	zinc finger protein 680	-0.495	1.25E-4
TTC13	tetratricopeptide repeat domain 13	-0.495	1.25E-4
ZDHHC13	zinc finger, DHHC-type containing 13	-0.495	1.26E-4
UBXN7	UBX domain protein 7	-0.495	1.26E-4
GPR52	G protein-coupled receptor 52	-0.495	1.27E-4
MYST4	MYST histone acetyltransferase (monocytic leukemia) 4	-0.495	1.27E-4
ANKRD16	ankyrin repeat domain 16	-0.495	1.28E-4
MYO5A	myosin VA (heavy chain 12, myoxin)	-0.495	1.28E-4
MLLT6	myeloid/lymphoid or mixed-lineage leukemia (trithorax homolog, Drosophila); translocated to, 6	-0.495	1.28E-4
TMEM48	transmembrane protein 48	-0.495	1.29E-4
PAPOLG	poly(A) polymerase gamma	-0.495	1.29E-4
TNIK	TRAF2 and NCK interacting kinase	-0.494	1.30E-4
ZNF263	zinc finger protein 263	-0.494	1.30E-4

Gene symbol	Gene title	ρ	Adjusted *P*
DHX16	DEAH (Asp-Glu-Ala-His) box polypeptide 16	-0.494	1.30E-4
STX17	syntaxin 17	-0.494	1.30E-4
ZBTB25	zinc finger and BTB domain containing 25	-0.494	1.31E-4
SNRNP48	small nuclear ribonucleoprotein 48kDa (U11/U12)	-0.494	1.31E-4
L3MBTL3	l(3)mbt-like 3 (Drosophila)	-0.494	1.31E-4
ATP8B2	ATPase, class I, type 8B, member 2	-0.494	1.31E-4
ZNF136	zinc finger protein 136	-0.494	1.32E-4
SRSF11	serine/arginine-rich splicing factor 11	-0.494	1.33E-4
ATP9B	ATPase, class II, type 9B	-0.494	1.33E-4
PAXIP1	PAX interacting (with transcription-activation domain) protein 1	-0.494	1.33E-4
C9orf156	chromosome 9 open reading frame 156	-0.493	1.33E-4
SMEK1	SMEK homolog 1, suppressor of mek1 (Dictyostelium)	-0.493	1.33E-4
HSPH1	heat shock 105kDa/110kDa protein 1	-0.493	1.33E-4
HINT1	histidine triad nucleotide binding protein 1	-0.493	1.34E-4
ATF7IP	activating transcription factor 7 interacting protein	-0.493	1.35E-4
CAMK4	calcium/calmodulin-dependent protein kinase IV	-0.493	1.35E-4
CELF1	CUGBP, Elav-like family member 1	-0.493	1.35E-4
TXK	TXK tyrosine kinase	-0.493	1.36E-4
TNRC6A	trinucleotide repeat containing 6A	-0.493	1.36E-4
THADA	thyroid adenoma associated	-0.493	1.36E-4
MTO1	mitochondrial translation optimization 1 homolog (S. cerevisiae)	-0.493	1.37E-4
ERCC5	excision repair cross-complementing rodent repair deficiency, complementation group 5	-0.493	1.37E-4
NOL8	nucleolar protein 8	-0.492	1.37E-4
REV1	REV1 homolog (S. cerevisiae)	-0.492	1.37E-4
C7orf36	chromosome 7 open reading frame 36	-0.492	1.37E-4
ARMC1	armadillo repeat containing 1	-0.492	1.38E-4
HERC1	hect (homologous to the E6-AP (UBE3A) carboxyl terminus) domain and RCC1 (CHC1)-like domain (RLD) 1	-0.492	1.38E-4
RC3H2	ring finger and CCCH-type domains 2	-0.492	1.38E-4
DDX50	DEAD (Asp-Glu-Ala-Asp) box polypeptide 50	-0.492	1.38E-4
STX2	syntaxin 2	-0.492	1.38E-4
TRNT1	tRNA nucleotidyl transferase, CCA-adding, 1	-0.492	1.38E-4
CDC25B	cell division cycle 25 homolog B (S. pombe)	-0.492	1.38E-4
CASD1	CAS1 domain containing 1	-0.492	1.39E-4
CDK13	cyclin-dependent kinase 13	-0.492	1.39E-4
MAN1A2	mannosidase, alpha, class 1A, member 2	-0.492	1.39E-4
TUT1	terminal uridylyl transferase 1, U6 snRNA-specific	-0.492	1.39E-4
TRIM32	tripartite motif-containing 32	-0.492	1.39E-4
EIF2C3	eukaryotic translation initiation factor 2C, 3	-0.491	1.40E-4
TMEM19	transmembrane protein 19	-0.491	1.41E-4
USP53	ubiquitin specific peptidase 53	-0.491	1.41E-4
REV3L	REV3-like, catalytic subunit of DNA polymerase zeta (yeast)	-0.491	1.41E-4
CCDC138	coiled-coil domain containing 138	-0.491	1.41E-4
CD2	CD2 molecule	-0.491	1.42E-4
PDE7A	phosphodiesterase 7A	-0.491	1.42E-4
USP24	ubiquitin specific peptidase 24	-0.491	1.43E-4

Gene symbol	Gene title	ρ	Adjusted *P*
SART3	squamous cell carcinoma antigen recognized by T cells 3	-0.491	1.43E-4
C20orf112	chromosome 20 open reading frame 112	-0.491	1.43E-4
MGAT4A	mannosyl (alpha-1,3-)-glycoprotein beta-1,4-N-acetylglucosaminyltransferase, isozyme A	-0.491	1.43E-4
BBS9	Bardet-Biedl syndrome 9	-0.490	1.43E-4
ZNF529	zinc finger protein 529	-0.490	1.43E-4
TRAPPC4	trafficking protein particle complex 4	-0.490	1.43E-4
IPO5	importin 5	-0.490	1.43E-4
HUS1	HUS1 checkpoint homolog (S. pombe)	-0.490	1.44E-4
TRPC1	transient receptor potential cation channel, subfamily C, member 1	-0.490	1.44E-4
RABEP1	rabaptin, RAB GTPase binding effector protein 1	-0.490	1.45E-4
BUD13	BUD13 homolog (S. cerevisiae)	-0.490	1.45E-4
DET1	de-etiolated homolog 1 (Arabidopsis)	-0.490	1.45E-4
SPECC1L	sperm antigen with calponin homology and coiled-coil domains 1-like	-0.490	1.45E-4
ZFYVE27	zinc finger, FYVE domain containing 27	-0.490	1.46E-4
MRPL50	mitochondrial ribosomal protein L50	-0.490	1.46E-4
EPM2AIP1	EPM2A (laforin) interacting protein 1	-0.490	1.46E-4
NAPEPLD	N-acyl phosphatidylethanolamine phospholipase D	-0.490	1.46E-4
GIMAP6	GTPase, IMAP family member 6	-0.490	1.46E-4
GGA2	golgi-associated, gamma adaptin ear containing, ARF binding protein 2	-0.490	1.46E-4
RNF144A	ring finger protein 144A	-0.490	1.46E-4
USP20	ubiquitin specific peptidase 20	-0.489	1.48E-4
KDM4A	lysine (K)-specific demethylase 4A	-0.489	1.48E-4
LUC7L	LUC7-like (S. cerevisiae)	-0.489	1.48E-4
LEO1	Leo1, Paf1/RNA polymerase II complex component, homolog (S. cerevisiae)	-0.489	1.48E-4
BBS7	Bardet-Biedl syndrome 7	-0.489	1.49E-4
SCAPER	S-phase cyclin A-associated protein in the ER	-0.489	1.49E-4
IARS	isoleucyl-tRNA synthetase	-0.489	1.49E-4
ZHX2	zinc fingers and homeoboxes 2	-0.489	1.50E-4
AKAP11	A kinase (PRKA) anchor protein 11	-0.489	1.51E-4
KCNA3	potassium voltage-gated channel, shaker-related subfamily, member 3	-0.489	1.51E-4
POLR3B	polymerase (RNA) III (DNA directed) polypeptide B	-0.488	1.52E-4
KDM5A	lysine (K)-specific demethylase 5A	-0.488	1.53E-4
GLT8D1	glycosyltransferase 8 domain containing 1	-0.488	1.53E-4
ABI2	abl-interactor 2	-0.488	1.53E-4
ENTPD4	ectonucleoside triphosphate diphosphohydrolase 4	-0.488	1.54E-4
PAAF1	proteasomal ATPase-associated factor 1	-0.488	1.54E-4
TTC1	tetratricopeptide repeat domain 1	-0.488	1.54E-4
ESCO1	establishment of cohesion 1 homolog 1 (S. cerevisiae)	-0.488	1.54E-4
DTNB	dystrobrevin, beta	-0.488	1.54E-4
SETD5	SET domain containing 5	-0.488	1.54E-4
PIGC	phosphatidylinositol glycan anchor biosynthesis, class C	-0.488	1.54E-4
ZNF445	zinc finger protein 445	-0.488	1.54E-4
TCTN1	tectonic family member 1	-0.488	1.55E-4

Gene symbol	Gene title	ρ	Adjusted P
EIF2B5	eukaryotic translation initiation factor 2B, subunit 5 epsilon, 82kDa	-0.488	1.55E-4
NAE1	NEDD8 activating enzyme E1 subunit 1	-0.488	1.55E-4
SUPT3H	suppressor of Ty 3 homolog (S. cerevisiae)	-0.487	1.55E-4
MALT1	mucosa associated lymphoid tissue lymphoma translocation gene 1	-0.487	1.55E-4
CCDC82	coiled-coil domain containing 82	-0.487	1.55E-4
SMARCA2	SWI/SNF related, matrix associated, actin dependent regulator of chromatin, subfamily a, member 2	-0.487	1.56E-4
ADAM22	ADAM metallopeptidase domain 22	-0.487	1.56E-4
NUP155	nucleoporin 155kDa	-0.487	1.56E-4
ZNF192	zinc finger protein 192	-0.487	1.56E-4
HSF2	heat shock transcription factor 2	-0.487	1.56E-4
SHQ1	SHQ1 homolog (S. cerevisiae)	-0.487	1.57E-4
SLC25A17	solute carrier family 25 (mitochondrial carrier; peroxisomal membrane protein, 34kDa), member 17	-0.487	1.58E-4
GTPBP8	GTP-binding protein 8 (putative)	-0.487	1.59E-4
SRPRB	signal recognition particle receptor, B subunit	-0.487	1.59E-4
AGFG2	ArfGAP with FG repeats 2	-0.487	1.59E-4
TMEM106B	transmembrane protein 106B	-0.487	1.59E-4
POLR3H	polymerase (RNA) III (DNA directed) polypeptide H (22.9kD)	-0.486	1.59E-4
STYX	serine/threonine/tyrosine interacting protein	-0.486	1.59E-4
CARKD	carbohydrate kinase domain containing	-0.486	1.59E-4
ACVR2A	activin A receptor, type IIA	-0.486	1.59E-4
PRRC1	proline-rich coiled-coil 1	-0.486	1.59E-4
INO80D	INO80 complex subunit D	-0.486	1.59E-4
RUFY2	RUN and FYVE domain containing 2	-0.486	1.60E-4
MTERFD3	MTERF domain containing 3	-0.486	1.60E-4
ZNF549	zinc finger protein 549	-0.486	1.60E-4
CCDC123	coiled-coil domain containing 123	-0.486	1.60E-4
FAM120B	family with sequence similarity 120B	-0.486	1.61E-4
OVGP1	oviductal glycoprotein 1, 120kDa	-0.486	1.61E-4
TDP1	tyrosyl-DNA phosphodiesterase 1	-0.486	1.61E-4
RNF121	ring finger protein 121	-0.486	1.62E-4
TUBD1	tubulin, delta 1	-0.486	1.62E-4
SKP2	S-phase kinase-associated protein 2 (p45)	-0.485	1.63E-4
YTHDC1	YTH domain containing 1	-0.485	1.63E-4
EDC3	enhancer of mRNA decapping 3 homolog (S. cerevisiae)	-0.485	1.64E-4
SMARCAD1	SWI/SNF-related, matrix-associated actin-dependent regulator of chromatin, subfamily a, containing DEAD/H box 1	-0.485	1.64E-4
UBR1	ubiquitin protein ligase E3 component n-recognin 1	-0.485	1.64E-4
ZNF827	zinc finger protein 827	-0.485	1.65E-4
C3orf75	chromosome 3 open reading frame 75	-0.485	1.65E-4
ESYT1	extended synaptotagmin-like protein 1	-0.485	1.66E-4
KLHL20	kelch-like 20 (Drosophila)	-0.485	1.66E-4
RBM19	RNA binding motif protein 19	-0.485	1.66E-4
UTP15	UTP15, U3 small nucleolar ribonucleoprotein, homolog (S. cerevisiae)	-0.485	1.66E-4

Gene symbol	Gene title	ρ	Adjusted *P*
KIAA1919	KIAA1919	-0.485	1.66E-4
TMEM14A	transmembrane protein 14A	-0.485	1.66E-4
FBXO8	F-box protein 8	-0.485	1.66E-4
CCDC14	coiled-coil domain containing 14	-0.484	1.68E-4
FOXP1	forkhead box P1	-0.484	1.68E-4
ASF1A	ASF1 anti-silencing function 1 homolog A (S. cerevisiae)	-0.484	1.69E-4
JAK1	Janus kinase 1	-0.484	1.69E-4
BIRC6	baculoviral IAP repeat-containing 6	-0.484	1.70E-4
KDM3A	lysine (K)-specific demethylase 3A	-0.484	1.70E-4
RNASEH2B	ribonuclease H2, subunit B	-0.484	1.70E-4
FASLG	Fas ligand (TNF superfamily, member 6)	-0.484	1.70E-4
DNMT3A	DNA (cytosine-5-)-methyltransferase 3 alpha	-0.484	1.71E-4
HSPA1L	heat shock 70kDa protein 1-like	-0.484	1.71E-4
ZNF770	zinc finger protein 770	-0.484	1.72E-4
C15orf17	chromosome 15 open reading frame 17	-0.484	1.72E-4
XKR6	XK, Kell blood group complex subunit-related family, member 6	-0.484	1.72E-4
HOXB2	homeobox B2	-0.483	1.73E-4
GZMK	granzyme K (granzyme 3; tryptase II)	-0.483	1.73E-4
BLMH	bleomycin hydrolase	-0.483	1.73E-4
MPHOSPH8	M-phase phosphoprotein 8	-0.483	1.73E-4
UBA5	ubiquitin-like modifier activating enzyme 5	-0.483	1.73E-4
PCSK7	proprotein convertase subtilisin/kexin type 7	-0.483	1.73E-4
TULP4	tubby like protein 4	-0.483	1.73E-4
ATIC	5-aminoimidazole-4-carboxamide ribonucleotide formyltransferase/IMP cyclohydrolase	-0.483	1.75E-4
CCDC77	coiled-coil domain containing 77	-0.483	1.75E-4
CBLB	Cas-Br-M (murine) ecotropic retroviral transforming sequence b	-0.483	1.75E-4
C2orf44	chromosome 2 open reading frame 44	-0.483	1.76E-4
PHF3	PHD finger protein 3	-0.483	1.77E-4
AMMECR1L	AMME chromosomal region gene 1-like	-0.483	1.77E-4
ARHGAP5	Rho GTPase activating protein 5	-0.483	1.77E-4
TIGIT	T cell immunoreceptor with Ig and ITIM domains	-0.482	1.79E-4
FN3KRP	fructosamine 3 kinase related protein	-0.482	1.79E-4
PPAT	phosphoribosyl pyrophosphate amidotransferase	-0.482	1.79E-4
HSPA14	heat shock 70kDa protein 14	-0.482	1.79E-4
C17orf42	chromosome 17 open reading frame 42	-0.482	1.79E-4
RHOH	ras homolog gene family, member H	-0.482	1.80E-4
ZNF25	zinc finger protein 25	-0.482	1.80E-4
UBE3B	ubiquitin protein ligase E3B	-0.482	1.81E-4
PPP2R5C	protein phosphatase 2, regulatory subunit B', gamma	-0.482	1.81E-4
GLS	glutaminase	-0.482	1.81E-4
KLHDC2	kelch domain containing 2	-0.482	1.81E-4
RCN2	reticulocalbin 2, EF-hand calcium binding domain	-0.482	1.82E-4
PDS5A	PDS5, regulator of cohesion maintenance, homolog A (S. cerevisiae)	-0.482	1.82E-4
NOP14	NOP14 nucleolar protein homolog (yeast)	-0.482	1.82E-4
OBFC1	oligonucleotide/oligosaccharide-binding fold containing 1	-0.482	1.82E-4

Gene symbol	Gene title	ρ	Adjusted *P*
POLA2	polymerase (DNA directed), alpha 2 (70kD subunit)	-0.481	1.83E-4
ZNHIT3	zinc finger, HIT-type containing 3	-0.481	1.85E-4
VPS13B	vacuolar protein sorting 13 homolog B (yeast)	-0.481	1.85E-4
MTERF	mitochondrial transcription termination factor	-0.481	1.85E-4
CSE1L	CSE1 chromosome segregation 1-like (yeast)	-0.481	1.85E-4
RNF2	ring finger protein 2	-0.481	1.85E-4
IL2RB	interleukin 2 receptor, beta	-0.481	1.85E-4
ACACA	acetyl-CoA carboxylase alpha	-0.481	1.85E-4
CYLD	cylindromatosis (turban tumor syndrome)	-0.481	1.86E-4
TGFBR3	transforming growth factor, beta receptor III	-0.481	1.86E-4
POGZ	pogo transposable element with ZNF domain	-0.481	1.86E-4
MFSD11	major facilitator superfamily domain containing 11	-0.481	1.86E-4
PDP2	pyruvate dehyrogenase phosphatase catalytic subunit 2	-0.480	1.87E-4
TMEM168	transmembrane protein 168	-0.480	1.88E-4
LOC100128816	ACAH3104	-0.480	1.88E-4
NCAPD3	non-SMC condensin II complex, subunit D3	-0.480	1.88E-4
NAA38	N(alpha)-acetyltransferase 38, NatC auxiliary subunit	-0.480	1.89E-4
ASXL2	additional sex combs like 2 (Drosophila)	-0.480	1.89E-4
PEX3	peroxisomal biogenesis factor 3	-0.480	1.89E-4
ATP6V1H	ATPase, H+ transporting, lysosomal 50/57kDa, V1 subunit H	-0.480	1.91E-4
ZCCHC8	zinc finger, CCHC domain containing 8	-0.480	1.91E-4
CLPX	ClpX caseinolytic peptidase X homolog (E. coli)	-0.480	1.91E-4
EXOSC2	exosome component 2	-0.480	1.92E-4
LRBA	LPS-responsive vesicle trafficking, beach and anchor containing	-0.480	1.92E-4
MON2	MON2 homolog (S. cerevisiae)	-0.479	1.93E-4
APBA2	amyloid beta (A4) precursor protein-binding, family A, member 2	-0.479	1.94E-4
TC2N	tandem C2 domains, nuclear	-0.479	1.94E-4
SUPT6H	suppressor of Ty 6 homolog (S. cerevisiae)	-0.479	1.96E-4
KIAA1429	KIAA1429	-0.479	1.97E-4
SDHA	succinate dehydrogenase complex, subunit A, flavoprotein (Fp)	-0.479	1.97E-4
THOC1	THO complex 1	-0.479	1.97E-4
INTS2	integrator complex subunit 2	-0.478	1.99E-4
NFYB	nuclear transcription factor Y, beta	-0.478	1.99E-4
ATXN10	ataxin 10	-0.478	1.99E-4
SEC61A2	Sec61 alpha 2 subunit (S. cerevisiae)	-0.478	1.99E-4
DCP1B	DCP1 decapping enzyme homolog B (S. cerevisiae)	-0.478	2.00E-4
DHX35	DEAH (Asp-Glu-Ala-His) box polypeptide 35	-0.478	2.00E-4
EIF4ENIF1	eukaryotic translation initiation factor 4E nuclear import factor 1	-0.478	2.00E-4
BCL11B	B-cell CLL/lymphoma 11B (zinc finger protein)	-0.478	2.01E-4
THAP6	THAP domain containing 6	-0.478	2.01E-4
ZFYVE9	zinc finger, FYVE domain containing 9	-0.478	2.01E-4
SUGT1	SGT1, suppressor of G2 allele of SKP1 (S. cerevisiae)	-0.478	2.01E-4
TTC19	tetratricopeptide repeat domain 19	-0.478	2.01E-4
SEC22C	SEC22 vesicle trafficking protein homolog C (S. cerevisiae)	-0.478	2.01E-4
PWP2	PWP2 periodic tryptophan protein homolog (yeast)	-0.478	2.01E-4

Gene symbol	Gene title	ρ	Adjusted *P*
MYST2	MYST histone acetyltransferase 2	-0.478	2.01E-4
FANCD2	Fanconi anemia, complementation group D2	-0.478	2.01E-4
TTC21B	tetratricopeptide repeat domain 21B	-0.478	2.01E-4
ZKSCAN1	zinc finger with KRAB and SCAN domains 1	-0.478	2.01E-4
ZNF398	zinc finger protein 398	-0.478	2.02E-4
POLR3E	polymerase (RNA) III (DNA directed) polypeptide E (80kD)	-0.478	2.02E-4
SCML4	sex comb on midleg-like 4 (Drosophila)	-0.478	2.02E-4
SPOCK2	sparc/osteonectin, cwcv and kazal-like domains proteoglycan (testican) 2	-0.478	2.02E-4
PHF14	PHD finger protein 14	-0.478	2.02E-4
C14orf104	chromosome 14 open reading frame 104	-0.478	2.02E-4
USP34	ubiquitin specific peptidase 34	-0.477	2.03E-4
BBS4	Bardet-Biedl syndrome 4	-0.477	2.03E-4
SEPT1	septin 1	-0.477	2.04E-4
TMEM107	transmembrane protein 107	-0.477	2.04E-4
ZNF224	zinc finger protein 224	-0.477	2.06E-4
THNSL1	threonine synthase-like 1 (S. cerevisiae)	-0.477	2.07E-4
FZD3	frizzled homolog 3 (Drosophila)	-0.477	2.07E-4
ZBTB39	zinc finger and BTB domain containing 39	-0.477	2.07E-4
ICK	intestinal cell (MAK-like) kinase	-0.477	2.09E-4
PITRM1	pitrilysin metallopeptidase 1	-0.476	2.10E-4
SFSWAP	splicing factor, suppressor of white-apricot homolog (Drosophila)	-0.476	2.11E-4
WDR5B	WD repeat domain 5B	-0.476	2.11E-4
KRI1	KRI1 homolog (S. cerevisiae)	-0.476	2.13E-4
DDX24	DEAD (Asp-Glu-Ala-Asp) box polypeptide 24	-0.476	2.13E-4
NSUN6	NOP2/Sun domain family, member 6	-0.476	2.14E-4
MAPK8	mitogen-activated protein kinase 8	-0.476	2.14E-4
CCDC93	coiled-coil domain containing 93	-0.476	2.14E-4
RFT1	RFT1 homolog (S. cerevisiae)	-0.475	2.15E-4
RYK	RYK receptor-like tyrosine kinase	-0.475	2.15E-4
MMS22L	MMS22-like, DNA repair protein	-0.475	2.15E-4
USP44	ubiquitin specific peptidase 44	-0.475	2.18E-4
MMS19	MMS19 nucleotide excision repair homolog (S. cerevisiae)	-0.475	2.18E-4
BPTF	bromodomain PHD finger transcription factor	-0.475	2.22E-4
ZNF175	zinc finger protein 175	-0.474	2.23E-4
ZNF512	zinc finger protein 512	-0.474	2.23E-4
FBXL4	F-box and leucine-rich repeat protein 4	-0.474	2.23E-4
TNKS	tankyrase, TRF1-interacting ankyrin-related ADP-ribose polymerase	-0.474	2.26E-4
C14orf135	chromosome 14 open reading frame 135	-0.474	2.27E-4
AKAP10	A kinase (PRKA) anchor protein 10	-0.474	2.27E-4
ZNF589	zinc finger protein 589	-0.474	2.27E-4
C5orf34	chromosome 5 open reading frame 34	-0.474	2.29E-4
RCOR3	REST corepressor 3	-0.474	2.29E-4
GPR155	G protein-coupled receptor 155	-0.473	2.30E-4
PAN2	PAN2 poly(A) specific ribonuclease subunit homolog (S. cerevisiae)	-0.473	2.30E-4
POT1	protection of telomeres 1 homolog (S. pombe)	-0.473	2.31E-4

Gene symbol	Gene title	ρ	Adjusted P
ASNS	asparagine synthetase (glutamine-hydrolyzing)	-0.473	2.31E-4
SAMD3	sterile alpha motif domain containing 3	-0.473	2.31E-4
HARS	histidyl-tRNA synthetase	-0.473	2.33E-4
PRPF4	PRP4 pre-mRNA processing factor 4 homolog (yeast)	-0.473	2.34E-4
ZNF131	zinc finger protein 131	-0.473	2.34E-4
MYO1D	myosin ID	-0.473	2.34E-4
WDR36	WD repeat domain 36	-0.473	2.35E-4
CDK5RAP2	CDK5 regulatory subunit associated protein 2	-0.473	2.35E-4
ATR	ataxia telangiectasia and Rad3 related	-0.472	2.37E-4
PRPF6	PRP6 pre-mRNA processing factor 6 homolog (S. cerevisiae)	-0.472	2.37E-4
ZNF555	zinc finger protein 555	-0.472	2.40E-4
HOOK1	hook homolog 1 (Drosophila)	-0.472	2.40E-4
NIPAL2	NIPA-like domain containing 2	-0.472	2.40E-4
TNRC6B	trinucleotide repeat containing 6B	-0.472	2.41E-4
XPO5	exportin 5	-0.472	2.41E-4
MRPL1	mitochondrial ribosomal protein L1	-0.472	2.41E-4
POLR2H	polymerase (RNA) II (DNA directed) polypeptide H	-0.472	2.41E-4
MRPS31	mitochondrial ribosomal protein S31	-0.472	2.43E-4
ZCCHC4	zinc finger, CCHC domain containing 4	-0.471	2.45E-4
MRPS25	mitochondrial ribosomal protein S25	-0.471	2.45E-4
SENP7	SUMO1/sentrin specific peptidase 7	-0.471	2.47E-4
MAP3K4	mitogen-activated protein kinase kinase kinase 4	-0.471	2.47E-4
METT11D1	methyltransferase 11 domain containing 1	-0.471	2.48E-4
ZNF276	zinc finger protein 276	-0.471	2.48E-4
SC4MOL	sterol-C4-methyl oxidase-like	-0.471	2.49E-4
PMS1	PMS1 postmeiotic segregation increased 1 (S. cerevisiae)	-0.471	2.49E-4
C12orf66	chromosome 12 open reading frame 66	-0.471	2.49E-4
CBFA2T2	core-binding factor, runt domain, alpha subunit 2; translocated to, 2	-0.470	2.50E-4
NAPB	N-ethylmaleimide-sensitive factor attachment protein, beta	-0.470	2.50E-4
ALS2CR8	amyotrophic lateral sclerosis 2 (juvenile) chromosome region, candidate 8	-0.470	2.50E-4
IL6ST	interleukin 6 signal transducer (gp130, oncostatin M receptor)	-0.470	2.52E-4
ZNF256	zinc finger protein 256	-0.470	2.52E-4
CCND2	cyclin D2	-0.470	2.52E-4
CD6	CD6 molecule	-0.470	2.54E-4
C21orf59	chromosome 21 open reading frame 59	-0.470	2.54E-4
GSDMB	gasdermin B	-0.470	2.56E-4
C9orf95	chromosome 9 open reading frame 95	-0.470	2.58E-4
ZNF189	zinc finger protein 189	-0.469	2.59E-4
GTF2E1	general transcription factor IIE, polypeptide 1, alpha 56kDa	-0.469	2.59E-4
YLPM1	YLP motif containing 1	-0.469	2.61E-4
C14orf93	chromosome 14 open reading frame 93	-0.469	2.61E-4
SPTBN1	spectrin, beta, non-erythrocytic 1	-0.469	2.61E-4
USP45	ubiquitin specific peptidase 45	-0.469	2.64E-4
KRIT1	KRIT1, ankyrin repeat containing	-0.469	2.64E-4
CDC23	cell division cycle 23 homolog (S. cerevisiae)	-0.469	2.64E-4
SLC35A5	solute carrier family 35, member A5	-0.469	2.66E-4
EOMES	eomesodermin	-0.469	2.66E-4

Gene symbol	Gene title	ρ	Adjusted P
UBTF	upstream binding transcription factor, RNA polymerase I	-0.468	2.68E-4
ZCCHC14	zinc finger, CCHC domain containing 14	-0.468	2.68E-4
C9orf41	chromosome 9 open reading frame 41	-0.468	2.70E-4
MYNN	myoneurin	-0.468	2.70E-4
MTOR	mechanistic target of rapamycin (serine/threonine kinase)	-0.468	2.70E-4
TTC39B	tetratricopeptide repeat domain 39B	-0.468	2.71E-4
CEP57	centrosomal protein 57kDa	-0.468	2.71E-4
C17orf75	chromosome 17 open reading frame 75	-0.468	2.71E-4
HNRPDL	heterogeneous nuclear ribonucleoprotein D-like	-0.468	2.72E-4
FBXO4	F-box protein 4	-0.468	2.72E-4
ZNF619	zinc finger protein 619	-0.468	2.72E-4
SMCR8	Smith-Magenis syndrome chromosome region, candidate 8	-0.468	2.72E-4
ZBTB44	zinc finger and BTB domain containing 44	-0.468	2.73E-4
COG3	component of oligomeric golgi complex 3	-0.468	2.73E-4
PLK1S1	polo-like kinase 1 substrate 1	-0.468	2.73E-4
LRPPRC	leucine-rich PPR-motif containing	-0.468	2.73E-4
GAN	gigaxonin	-0.468	2.73E-4
ZMYND8	zinc finger, MYND-type containing 8	-0.468	2.73E-4
RPAIN	RPA interacting protein	-0.467	2.75E-4
CETN3	centrin, EF-hand protein, 3	-0.467	2.76E-4
EHBP1	EH domain binding protein 1	-0.467	2.76E-4
RAD54L2	RAD54-like 2 (S. cerevisiae)	-0.467	2.76E-4
EDC4	enhancer of mRNA decapping 4	-0.467	2.78E-4
PDE3B	phosphodiesterase 3B, cGMP-inhibited	-0.467	2.78E-4
ZW10	ZW10, kinetochore associated, homolog (Drosophila)	-0.467	2.78E-4
FAM116A	family with sequence similarity 116, member A	-0.467	2.78E-4
THEM4	thioesterase superfamily member 4	-0.467	2.79E-4
NBAS	neuroblastoma amplified sequence	-0.467	2.79E-4
ANKRD5	ankyrin repeat domain 5	-0.467	2.79E-4
IPO9	importin 9	-0.467	2.80E-4
TPR	translocated promoter region (to activated MET oncogene)	-0.467	2.80E-4
VPS13A	vacuolar protein sorting 13 homolog A (S. cerevisiae)	-0.467	2.80E-4
CCT3	chaperonin containing TCP1, subunit 3 (gamma)	-0.467	2.80E-4
VPS72	vacuolar protein sorting 72 homolog (S. cerevisiae)	-0.467	2.80E-4
FANCC	Fanconi anemia, complementation group C	-0.466	2.82E-4
ANKRA2	ankyrin repeat, family A (RFXANK-like), 2	-0.466	2.82E-4
TTC27	tetratricopeptide repeat domain 27	-0.466	2.82E-4
PIKFYVE	phosphoinositide kinase, FYVE finger containing	-0.466	2.82E-4
RASGRP2	RAS guanyl releasing protein 2 (calcium and DAG-regulated)	-0.466	2.82E-4
C1orf25	chromosome 1 open reading frame 25	-0.466	2.83E-4
C5orf53	chromosome 5 open reading frame 53	-0.466	2.83E-4
ANKRD44	ankyrin repeat domain 44	-0.466	2.83E-4
UFSP2	UFM1-specific peptidase 2	-0.466	2.83E-4
C21orf96	chromosome 21 open reading frame 96	-0.466	2.84E-4
DCUN1D5	DCN1, defective in cullin neddylation 1, domain containing 5 (S. cerevisiae)	-0.466	2.84E-4
PFKM	phosphofructokinase, muscle	-0.466	2.84E-4
C1orf107	chromosome 1 open reading frame 107	-0.466	2.84E-4
WWP1	WW domain containing E3 ubiquitin protein ligase 1	-0.466	2.84E-4

Gene symbol	Gene title	*ρ*	Adjusted *P*
C12orf11	chromosome 12 open reading frame 11	-0.466	2.84E-4
PPIE	peptidylprolyl isomerase E (cyclophilin E)	-0.466	2.88E-4
COX18	COX18 cytochrome c oxidase assembly homolog (S. cerevisiae)	-0.466	2.88E-4
ZNF266	zinc finger protein 266	-0.465	2.89E-4
UST	uronyl-2-sulfotransferase	-0.465	2.89E-4
KLHL11	kelch-like 11 (Drosophila)	-0.465	2.89E-4
TULP3	tubby like protein 3	-0.465	2.89E-4
C11orf31	chromosome 11 open reading frame 31	-0.465	2.92E-4
NUDCD3	NudC domain containing 3	-0.465	2.92E-4
XPO4	exportin 4	-0.465	2.93E-4
ZNF644	zinc finger protein 644	-0.465	2.93E-4
ZNF607	zinc finger protein 607	-0.465	2.93E-4
C12orf49	chromosome 12 open reading frame 49	-0.465	2.95E-4
HCFC2	host cell factor C2	-0.465	2.95E-4
ZNF828	zinc finger protein 828	-0.465	2.95E-4
MED1	mediator complex subunit 1	-0.465	2.95E-4
C5orf42	chromosome 5 open reading frame 42	-0.465	2.95E-4
C3orf63	chromosome 3 open reading frame 63	-0.464	2.96E-4
ZSCAN12	zinc finger and SCAN domain containing 12	-0.464	2.96E-4
SLC35B4	solute carrier family 35, member B4	-0.464	2.96E-4
DAP3	death associated protein 3	-0.464	2.96E-4
RCAN3	RCAN family member 3	-0.464	2.98E-4
C5orf39	chromosome 5 open reading frame 39	-0.464	2.98E-4
ANGEL1	angel homolog 1 (Drosophila)	-0.464	2.98E-4
ORC3	origin recognition complex, subunit 3	-0.464	2.98E-4
C9orf103	chromosome 9 open reading frame 103	-0.464	3.00E-4
GOLGA4	golgin A4	-0.464	3.00E-4
MKLN1	muskelin 1, intracellular mediator containing kelch motifs	-0.464	3.00E-4
SNX19	sorting nexin 19	-0.464	3.02E-4
MOCS2	molybdenum cofactor synthesis 2	-0.464	3.02E-4
MARCH9	membrane-associated ring finger (C3HC4) 9	-0.464	3.02E-4
CCDC25	coiled-coil domain containing 25	-0.464	3.03E-4
TIGD1	tigger transposable element derived 1	-0.464	3.03E-4
ANKS6	ankyrin repeat and sterile alpha motif domain containing 6	-0.464	3.03E-4
GOSR2	golgi SNAP receptor complex member 2	-0.464	3.03E-4
ZNF346	zinc finger protein 346	-0.464	3.03E-4
WDR27	WD repeat domain 27	-0.463	3.04E-4
IFT122	intraflagellar transport 122 homolog (Chlamydomonas)	-0.463	3.04E-4
FBXW11	F-box and WD repeat domain containing 11	-0.463	3.04E-4
TMEM263	Transmembrane Protein 263	-0.463	3.06E-4
EXOSC8	exosome component 8	-0.463	3.06E-4
GNG2	guanine nucleotide binding protein (G protein), gamma 2	-0.463	3.08E-4
ENO2	enolase 2 (gamma, neuronal)	-0.463	3.08E-4
ANKRD13C	ankyrin repeat domain 13C	-0.463	3.08E-4
SH3YL1	SH3 domain containing, Ysc84-like 1 (S. cerevisiae)	-0.463	3.09E-4
ZNF79	zinc finger protein 79	-0.463	3.09E-4
UBR5	ubiquitin protein ligase E3 component n-recognin 5	-0.463	3.09E-4
PIK3CA	phosphoinositide-3-kinase, catalytic, alpha polypeptide	-0.463	3.11E-4

Gene symbol	Gene title	ρ	Adjusted *P*
LDHB	lactate dehydrogenase B	-0.463	3.11E-4
ZNF418	zinc finger protein 418	-0.463	3.11E-4
USP16	ubiquitin specific peptidase 16	-0.463	3.11E-4
C15orf44	chromosome 15 open reading frame 44	-0.463	3.11E-4
CBLL1	Cas-Br-M (murine) ecotropic retroviral transforming sequence-like 1	-0.462	3.13E-4
MIS12	MIS12, MIND kinetochore complex component, homolog (S. pombe)	-0.462	3.15E-4
TARBP1	TAR (HIV-1) RNA binding protein 1	-0.462	3.15E-4
RALGPS2	Ral GEF with PH domain and SH3 binding motif 2	-0.462	3.15E-4
CCDC65	coiled-coil domain containing 65	-0.462	3.18E-4
PHC3	polyhomeotic homolog 3 (Drosophila)	-0.462	3.18E-4
CFL2	cofilin 2 (muscle)	-0.462	3.20E-4
WDR67	WD repeat domain 67	-0.462	3.20E-4
MTA2	metastasis associated 1 family, member 2	-0.462	3.20E-4
RBBP6	retinoblastoma binding protein 6	-0.461	3.24E-4
NARG2	NMDA receptor regulated 2	-0.461	3.24E-4
LARS	leucyl-tRNA synthetase	-0.461	3.24E-4
RRP1B	ribosomal RNA processing 1 homolog B (S. cerevisiae)	-0.461	3.25E-4
RPP30	ribonuclease P/MRP 30kDa subunit	-0.461	3.25E-4
DCAF16	DDB1 and CUL4 associated factor 16	-0.461	3.25E-4
C6orf192	chromosome 6 open reading frame 192	-0.461	3.25E-4
TRMU	tRNA 5-methylaminomethyl-2-thiouridylate methyltransferase	-0.461	3.25E-4
MAML2	mastermind-like 2 (Drosophila)	-0.461	3.27E-4
CHD2	chromodomain helicase DNA binding protein 2	-0.461	3.29E-4
DENR	density-regulated protein	-0.461	3.29E-4
PLEKHF1	pleckstrin homology domain containing, family F (with FYVE domain) member 1	-0.461	3.29E-4
UBR4	ubiquitin protein ligase E3 component n-recognin 4	-0.461	3.31E-4
BBX	bobby sox homolog (Drosophila)	-0.461	3.31E-4
TM7SF3	transmembrane 7 superfamily member 3	-0.461	3.31E-4
SEC24B	SEC24 family, member B (S. cerevisiae)	-0.460	3.32E-4
EFHC1	EF-hand domain (C-terminal) containing 1	-0.460	3.32E-4
SRSF7	serine/arginine-rich splicing factor 7	-0.460	3.32E-4
KIAA0090	KIAA0090	-0.460	3.32E-4
LASS5	LAG1 homolog, ceramide synthase 5	-0.460	3.34E-4
ANKZF1	ankyrin repeat and zinc finger domain containing 1	-0.460	3.34E-4
DNAJC16	DnaJ (Hsp40) homolog, subfamily C, member 16	-0.460	3.34E-4
STIM2	stromal interaction molecule 2	-0.460	3.34E-4
ARFGEF2	ADP-ribosylation factor guanine nucleotide-exchange factor 2 (brefeldin A-inhibited)	-0.460	3.36E-4
SACM1L	SAC1 suppressor of actin mutations 1-like (yeast)	-0.459	3.40E-4
NUP214	nucleoporin 214kDa	-0.459	3.40E-4
TMEM184C	transmembrane protein 184C	-0.459	3.40E-4
CEP70	centrosomal protein 70kDa	-0.459	3.40E-4
KLRAQ1	KLRAQ motif containing 1	-0.459	3.42E-4
NKTR	natural killer-tumor recognition sequence	-0.459	3.42E-4
ARIH2	ariadne homolog 2 (Drosophila)	-0.459	3.42E-4
FAM134B	family with sequence similarity 134, member B	-0.459	3.44E-4

Gene symbol	Gene title	ρ	Adjusted *P*
ZNF706	zinc finger protein 706	-0.459	3.44E-4
ELMOD2	ELMO/CED-12 domain containing 2	-0.459	3.44E-4
ATF2	activating transcription factor 2	-0.459	3.46E-4
AKD1	adenylate kinase domain containing 1	-0.459	3.46E-4
CD3E	CD3e molecule, epsilon (CD3-TCR complex)	-0.459	3.46E-4
MCM3	minichromosome maintenance complex component 3	-0.459	3.46E-4
ATAD1	ATPase family, AAA domain containing 1	-0.459	3.48E-4
SMYD4	SET and MYND domain containing 4	-0.459	3.48E-4
TAF1B	TATA box binding protein (TBP)-associated factor, RNA polymerase I, B, 63kDa	-0.459	3.48E-4
PRIM2	primase, DNA, polypeptide 2 (58kDa)	-0.459	3.48E-4
SLC30A6	solute carrier family 30 (zinc transporter), member 6	-0.459	3.48E-4
CD244	CD244 molecule, natural killer cell receptor 2B4	-0.458	3.50E-4
STK35	serine/threonine kinase 35	-0.458	3.50E-4
PPARD	peroxisome proliferator-activated receptor delta	-0.458	3.52E-4
SLC35F5	solute carrier family 35, member F5	-0.458	3.53E-4
ZNF532	zinc finger protein 532	-0.458	3.53E-4
SMYD2	SET and MYND domain containing 2	-0.458	3.53E-4
BTLA	B and T lymphocyte associated	-0.458	3.53E-4
GPR183	G protein-coupled receptor 183	-0.458	3.53E-4
GPR18	G protein-coupled receptor 18	-0.458	3.53E-4
FRYL	FRY-like	-0.458	3.55E-4
WDR19	WD repeat domain 19	-0.458	3.55E-4
EI24	etoposide induced 2.4 mRNA	-0.458	3.55E-4
TOP2B	topoisomerase (DNA) II beta 180kDa	-0.458	3.55E-4
SERINC5	serine incorporator 5	-0.458	3.56E-4
HDAC3	histone deacetylase 3	-0.458	3.56E-4
CXCR7	chemokine (C-X-C motif) receptor 7	-0.458	3.56E-4
SETDB2	SET domain, bifurcated 2	-0.457	3.58E-4
ZNF148	zinc finger protein 148	-0.457	3.58E-4
ZNF146	zinc finger protein 146	-0.457	3.58E-4
NSD1	nuclear receptor binding SET domain protein 1	-0.457	3.58E-4
ZBTB4	zinc finger and BTB domain containing 4	-0.457	3.58E-4
TM2D1	TM2 domain containing 1	-0.457	3.61E-4
ZNF782	zinc finger protein 782	-0.457	3.61E-4
SYNE1	spectrin repeat containing, nuclear envelope 1	-0.457	3.62E-4
GUF1	GUF1 GTPase homolog (S. cerevisiae)	-0.457	3.62E-4
DNAJC21	DnaJ (Hsp40) homolog, subfamily C, member 21	-0.457	3.62E-4
C14orf149	chromosome 14 open reading frame 149	-0.457	3.62E-4
SORCS3	sortilin-related VPS10 domain containing receptor 3	-0.457	3.62E-4
ETS1	v-ets erythroblastosis virus E26 oncogene homolog 1 (avian)	-0.457	3.64E-4
TNPO1	transportin 1	-0.457	3.64E-4
TBC1D4	TBC1 domain family, member 4	-0.457	3.64E-4
AASDH	aminoadipate-semialdehyde dehydrogenase	-0.457	3.64E-4
MRPL32	mitochondrial ribosomal protein L32	-0.457	3.64E-4
LIAS	lipoic acid synthetase	-0.457	3.65E-4
SATB1	SATB homeobox 1	-0.457	3.65E-4
MPP5	membrane protein, palmitoylated 5 (MAGUK p55 subfamily member 5)	-0.457	3.65E-4

Gene symbol	Gene title	ρ	Adjusted P
ATG5	ATG5 autophagy related 5 homolog (S. cerevisiae)	-0.457	3.65E-4
RNF168	ring finger protein 168	-0.457	3.65E-4
GTF2E2	general transcription factor IIE, polypeptide 2, beta 34kDa	-0.457	3.65E-4
ORC4	origin recognition complex, subunit 4	-0.457	3.65E-4
AGGF1	angiogenic factor with G patch and FHA domains 1	-0.456	3.67E-4
ZDHHC6	zinc finger, DHHC-type containing 6	-0.456	3.69E-4
WDR33	WD repeat domain 33	-0.456	3.69E-4
MPRIP	myosin phosphatase Rho interacting protein	-0.456	3.69E-4
TMEM63A	transmembrane protein 63A	-0.456	3.69E-4
RASA2	RAS p21 protein activator 2	-0.456	3.69E-4
GLG1	golgi glycoprotein 1	-0.456	3.73E-4
TPD52	tumor protein D52	-0.456	3.73E-4
CCDC45	coiled-coil domain containing 45	-0.456	3.73E-4
PCNX	pecanex homolog (Drosophila)	-0.456	3.73E-4
AHSA2	AHA1, activator of heat shock 90kDa protein ATPase homolog 2 (yeast)	-0.456	3.73E-4
G3BP1	GTPase activating protein (SH3 domain) binding protein 1	-0.456	3.73E-4
MAP2K5	mitogen-activated protein kinase kinase 5	-0.456	3.76E-4
SOD1	superoxide dismutase 1, soluble	-0.456	3.76E-4
PDK1	pyruvate dehydrogenase kinase, isozyme 1	-0.456	3.76E-4
PCNT	pericentrin	-0.456	3.76E-4
FASTKD1	FAST kinase domains 1	-0.455	3.78E-4
MCCC1	methylcrotonoyl-CoA carboxylase 1 (alpha)	-0.455	3.78E-4
C10orf57	chromosome 10 open reading frame 57	-0.455	3.81E-4
CPOX	coproporphyrinogen oxidase	-0.455	3.84E-4
PBRM1	polybromo 1	-0.455	3.84E-4
GNE	glucosamine (UDP-N-acetyl)-2-epimerase/N-acetylmannosamine kinase	-0.455	3.86E-4
CCDC104	coiled-coil domain containing 104	-0.455	3.86E-4
KIAA0753	KIAA0753	-0.455	3.86E-4
CREB1	cAMP responsive element binding protein 1	-0.454	3.89E-4
C5orf51	chromosome 5 open reading frame 51	-0.454	3.91E-4
HYLS1	hydrolethalus syndrome 1	-0.454	3.91E-4
LTN1	listerin E3 ubiquitin protein ligase 1	-0.454	3.91E-4
ZNF254	zinc finger protein 254	-0.454	3.91E-4
MTFMT	mitochondrial methionyl-tRNA formyltransferase	-0.454	3.91E-4
TRIP11	thyroid hormone receptor interactor 11	-0.454	3.94E-4
ZNF138	zinc finger protein 138	-0.454	3.96E-4
CHD1L	chromodomain helicase DNA binding protein 1-like	-0.454	3.96E-4
ACSS1	acyl-CoA synthetase short-chain family member 1	-0.454	3.96E-4
RBM16	RNA binding motif protein 16	-0.454	3.97E-4
OSBP	oxysterol binding protein	-0.454	3.97E-4
SDHAF2	succinate dehydrogenase complex assembly factor 2	-0.454	3.97E-4
ZNHIT6	zinc finger, HIT-type containing 6	-0.454	3.97E-4
CCR4	chemokine (C-C motif) receptor 4	-0.454	3.97E-4
APTX	aprataxin	-0.454	3.97E-4
ZNF236	zinc finger protein 236	-0.453	4.00E-4
TUBGCP4	tubulin, gamma complex associated protein 4	-0.453	4.03E-4
C1orf59	chromosome 1 open reading frame 59	-0.453	4.03E-4

Gene symbol	Gene title	ρ	Adjusted *P*
ZMYM2	zinc finger, MYM-type 2	-0.453	4.07E-4
POLR3K	polymerase (RNA) III (DNA directed) polypeptide K, 12.3 kDa	-0.453	4.07E-4
VCP	valosin-containing protein	-0.453	4.07E-4
TNFAIP8	tumor necrosis factor, alpha-induced protein 8	-0.453	4.07E-4
ALS2	amyotrophic lateral sclerosis 2 (juvenile)	-0.453	4.10E-4
RINT1	RAD50 interactor 1	-0.453	4.10E-4
GOLGA5	golgin A5	-0.452	4.13E-4
ABCF3	ATP-binding cassette, sub-family F (GCN20), member 3	-0.452	4.13E-4
SIKE1	suppressor of IKBKE 1	-0.452	4.15E-4
RAB7L1	RAB7, member RAS oncogene family-like 1	-0.452	4.15E-4
TRPM7	transient receptor potential cation channel, subfamily M, member 7	-0.452	4.15E-4
COG1	component of oligomeric golgi complex 1	-0.452	4.18E-4
TATDN2	TatD DNase domain containing 2	-0.452	4.18E-4
NAA35	N(alpha)-acetyltransferase 35, NatC auxiliary subunit	-0.452	4.20E-4
SECISBP2L	SECIS binding protein 2-like	-0.452	4.20E-4
ALMS1	Alstrom syndrome 1	-0.452	4.22E-4
ZBTB26	zinc finger and BTB domain containing 26	-0.452	4.22E-4
UHMK1	U2AF homology motif (UHM) kinase 1	-0.452	4.22E-4
MCOLN2	mucolipin 2	-0.452	4.22E-4
INVS	inversin	-0.451	4.25E-4
RSBN1L	round spermatid basic protein 1-like	-0.451	4.28E-4
CCDC132	coiled-coil domain containing 132	-0.451	4.31E-4
INTS8	integrator complex subunit 8	-0.451	4.34E-4
SETD2	SET domain containing 2	-0.451	4.34E-4
CD28	CD28 molecule	-0.451	4.34E-4
AAAS	achalasia, adrenocortical insufficiency, alacrimia	-0.451	4.37E-4
PCM1	pericentriolar material 1	-0.451	4.37E-4
CCDC134	coiled-coil domain containing 134	-0.450	4.39E-4
MYCBP2	MYC binding protein 2	-0.450	4.39E-4
UPF2	UPF2 regulator of nonsense transcripts homolog (yeast)	-0.450	4.39E-4
CEP135	centrosomal protein 135kDa	-0.450	4.39E-4
UVRAG	UV radiation resistance associated gene	-0.450	4.41E-4
DCP2	DCP2 decapping enzyme homolog (S. cerevisiae)	-0.450	4.41E-4
MPHOSPH10	M-phase phosphoprotein 10 (U3 small nucleolar ribonucleoprotein)	-0.450	4.41E-4
RIF1	RAP1 interacting factor homolog (yeast)	-0.450	4.41E-4
ARHGEF7	Rho guanine nucleotide exchange factor (GEF) 7	-0.450	4.41E-4
POLR1B	polymerase (RNA) I polypeptide B, 128kDa	-0.450	4.41E-4
SFXN1	sideroflexin 1	-0.450	4.41E-4
UGGT1	UDP-glucose glycoprotein glucosyltransferase 1	-0.450	4.43E-4
RPAP2	RNA polymerase II associated protein 2	-0.450	4.43E-4
SOAT1	sterol O-acyltransferase 1	-0.450	4.43E-4
FBXO3	F-box protein 3	-0.450	4.43E-4
RWDD2A	RWD domain containing 2A	-0.450	4.46E-4
OCIAD1	OCIA domain containing 1	-0.450	4.46E-4
C18orf55	chromosome 18 open reading frame 55	-0.450	4.46E-4
C18orf45	chromosome 18 open reading frame 45	-0.450	4.46E-4

Gene symbol	Gene title	ρ	Adjusted *P*
CBX5	chromobox homolog 5	-0.449	4.48E-4
DDX10	DEAD (Asp-Glu-Ala-Asp) box polypeptide 10	-0.449	4.48E-4
POLR2B	polymerase (RNA) II (DNA directed) polypeptide B, 140kDa	-0.449	4.48E-4
GGPS1	geranylgeranyl diphosphate synthase 1	-0.449	4.50E-4
FXR2	fragile X mental retardation, autosomal homolog 2	-0.449	4.50E-4
FAM40A	family with sequence similarity 40, member A	-0.449	4.50E-4
EXD2	exonuclease 3'-5' domain containing 2	-0.449	4.53E-4
DDX20	DEAD (Asp-Glu-Ala-Asp) box polypeptide 20	-0.449	4.53E-4
TMEM50B	transmembrane protein 50B	-0.449	4.56E-4
ZNF169	zinc finger protein 169	-0.449	4.56E-4
METTL3	methyltransferase like 3	-0.449	4.56E-4
ZCCHC11	zinc finger, CCHC domain containing 11	-0.449	4.59E-4
USP7	ubiquitin specific peptidase 7 (herpes virus-associated)	-0.449	4.59E-4
SGTB	small glutamine-rich tetratricopeptide repeat (TPR)-containing, beta	-0.449	4.59E-4
HELLS	helicase, lymphoid-specific	-0.448	4.62E-4
EPB41L5	erythrocyte membrane protein band 4.1 like 5	-0.448	4.62E-4
CCT4	chaperonin containing TCP1, subunit 4 (delta)	-0.448	4.65E-4
PSPC1	paraspeckle component 1	-0.448	4.65E-4
DMTF1	cyclin D binding myb-like transcription factor 1	-0.448	4.65E-4
CAMSAP1	calmodulin regulated spectrin-associated protein 1	-0.448	4.65E-4
NCALD	neurocalcin delta	-0.448	4.67E-4
ZNF304	zinc finger protein 304	-0.448	4.67E-4
PCF11	PCF11, cleavage and polyadenylation factor subunit, homolog (S. cerevisiae)	-0.448	4.67E-4
UBR3	ubiquitin protein ligase E3 component n-recognin 3 (putative)	-0.448	4.67E-4
ZAP70	zeta-chain (TCR) associated protein kinase 70kDa	-0.448	4.69E-4
C14orf102	chromosome 14 open reading frame 102	-0.448	4.69E-4
USP47	ubiquitin specific peptidase 47	-0.448	4.69E-4
AAGAB	alpha- and gamma-adaptin binding protein	-0.448	4.69E-4
PALB2	partner and localizer of BRCA2	-0.448	4.73E-4
AQR	aquarius homolog (mouse)	-0.448	4.73E-4
SYNE2	spectrin repeat containing, nuclear envelope 2	-0.448	4.73E-4
TCERG1	transcription elongation regulator 1	-0.447	4.74E-4
DLST	dihydrolipoamide S-succinyltransferase (E2 component of 2-oxo-glutarate complex)	-0.447	4.74E-4
C4orf21	chromosome 4 open reading frame 21	-0.447	4.74E-4
LEMD3	LEM domain containing 3	-0.447	4.74E-4
WDR91	WD repeat domain 91	-0.447	4.74E-4
PRKRA	protein kinase, interferon-inducible double stranded RNA dependent activator	-0.447	4.74E-4
PHF20	PHD finger protein 20	-0.447	4.74E-4
KIF3B	kinesin family member 3B	-0.447	4.77E-4
EBAG9	estrogen receptor binding site associated, antigen, 9	-0.447	4.77E-4
C21orf33	chromosome 21 open reading frame 33	-0.447	4.77E-4
MSH6	mutS homolog 6 (E. coli)	-0.447	4.80E-4
SPG7	spastic paraplegia 7 (pure and complicated autosomal recessive)	-0.447	4.80E-4
CTDSPL2	CTD (carboxy-terminal domain, RNA polymerase II,	-0.447	4.80E-4

Gene symbol	Gene title	ρ	Adjusted *P*
	polypeptide A) small phosphatase like 2		
CRTAM	cytotoxic and regulatory T cell molecule	-0.447	4.80E-4
ENOPH1	enolase-phosphatase 1	-0.447	4.82E-4
NRF1	nuclear respiratory factor 1	-0.447	4.82E-4
PRPF18	PRP18 pre-mRNA processing factor 18 homolog (S. cerevisiae)	-0.447	4.82E-4
MBNL2	muscleblind-like 2 (Drosophila)	-0.447	4.82E-4
EIF2AK3	eukaryotic translation initiation factor 2-alpha kinase 3	-0.447	4.82E-4
AEBP2	AE binding protein 2	-0.447	4.84E-4
ANKRD17	ankyrin repeat domain 17	-0.447	4.84E-4
DDI2	DNA-damage inducible 1 homolog 2 (S. cerevisiae)	-0.447	4.84E-4
UBE4B	ubiquitination factor E4B (UFD2 homolog, yeast)	-0.447	4.84E-4
ARCN1	archain 1	-0.447	4.84E-4
EXOSC1	exosome component 1	-0.447	4.84E-4
NSMCE4A	non-SMC element 4 homolog A (S. cerevisiae)	-0.447	4.84E-4
ARHGEF3	Rho guanine nucleotide exchange factor (GEF) 3	-0.446	4.87E-4
C12orf51	chromosome 12 open reading frame 51	-0.446	4.87E-4
HINFP	histone H4 transcription factor	-0.446	4.87E-4
AATF	apoptosis antagonizing transcription factor	-0.446	4.87E-4
RER1	RER1 retention in endoplasmic reticulum 1 homolog (S. cerevisiae)	-0.446	4.89E-4
ZNF107	zinc finger protein 107	-0.446	4.89E-4
MTMR2	myotubularin related protein 2	-0.446	4.89E-4
ACIN1	apoptotic chromatin condensation inducer 1	-0.446	4.89E-4
FAIM	Fas apoptotic inhibitory molecule	-0.446	4.96E-4
CCDC130	coiled-coil domain containing 130	-0.446	4.96E-4
MTA3	metastasis associated 1 family, member 3	-0.446	4.98E-4
CAND1	cullin-associated and neddylation-dissociated 1	-0.446	4.98E-4
C2orf56	chromosome 2 open reading frame 56	-0.446	4.98E-4
ZNF317	zinc finger protein 317	-0.446	4.98E-4
MRPS16	mitochondrial ribosomal protein S16	-0.446	4.98E-4
FHL2	four and a half LIM domains 2	0.446	4.98E-4
FGF18	fibroblast growth factor 18	0.446	4.96E-4
ADAMTS18	ADAM metallopeptidase with thrombospondin type 1 motif, 18	0.446	4.96E-4
GHRHR	growth hormone releasing hormone receptor	0.446	4.96E-4
HOXA3	homeobox A3	0.446	4.96E-4
ITGA7	integrin, alpha 7	0.446	4.93E-4
SLC6A13	solute carrier family 6 (neurotransmitter transporter, GABA), member 13	0.446	4.89E-4
PHACTR3	phosphatase and actin regulator 3	0.446	4.89E-4
MMD2	monocyte to macrophage differentiation-associated 2	0.447	4.82E-4
CDO1	cysteine dioxygenase, type I	0.447	4.82E-4
TAT	tyrosine aminotransferase	0.447	4.80E-4
RNASE1	ribonuclease, RNase A family, 1 (pancreatic)	0.447	4.77E-4
FAM43B	family with sequence similarity 43, member B	0.447	4.74E-4
ABCG8	ATP-binding cassette, sub-family G (WHITE), member 8	0.448	4.67E-4
MUC1	mucin 1, cell surface associated	0.448	4.67E-4
IRX5	iroquois homeobox 5	0.448	4.67E-4

Gene symbol	Gene title	ρ	Adjusted *P*
PPAP2C	phosphatidic acid phosphatase type 2C	0.448	4.65E-4
OPLAH	5-oxoprolinase (ATP-hydrolysing)	0.449	4.59E-4
GRHL3	grainyhead-like 3 (Drosophila)	0.449	4.53E-4
MAGI1	membrane associated guanylate kinase, WW and PDZ domain containing 1	0.449	4.53E-4
GPR61	G protein-coupled receptor 61	0.449	4.53E-4
ATP5G1	ATP synthase, H+ transporting, mitochondrial Fo complex, subunit C1 (subunit 9)	0.449	4.50E-4
ZNF541	zinc finger protein 541	0.449	4.50E-4
ADAMTS3	ADAM metallopeptidase with thrombospondin type 1 motif, 3	0.449	4.48E-4
DCLK1	doublecortin-like kinase 1	0.449	4.48E-4
LMX1A	LIM homeobox transcription factor 1, alpha	0.449	4.48E-4
PTCRA	pre T-cell antigen receptor alpha	0.450	4.46E-4
CCDC60	coiled-coil domain containing 60	0.450	4.43E-4
GRM7	glutamate receptor, metabotropic 7	0.450	4.41E-4
SEMA6B	sema domain, transmembrane domain (TM), and cytoplasmic domain, (semaphorin) 6B	0.451	4.37E-4
FAM53A	family with sequence similarity 53, member A	0.451	4.37E-4
SHROOM3	shroom family member 3	0.451	4.37E-4
CNGB1	cyclic nucleotide gated channel beta 1	0.451	4.28E-4
C2orf65	chromosome 2 open reading frame 65	0.451	4.28E-4
SLC25A34	solute carrier family 25, member 34	0.451	4.25E-4
SLC30A2	solute carrier family 30 (zinc transporter), member 2	0.451	4.25E-4
TNFRSF4	tumor necrosis factor receptor superfamily, member 4	0.452	4.22E-4
IFT140	intraflagellar transport 140 homolog (Chlamydomonas)	0.452	4.22E-4
DUSP13	dual specificity phosphatase 13	0.452	4.22E-4
ADAM9	ADAM metallopeptidase domain 9	0.452	4.20E-4
CAPN11	calpain 11	0.452	4.20E-4
CSMD2	CUB and Sushi multiple domains 2	0.452	4.15E-4
T	T, brachyury homolog (mouse)	0.452	4.15E-4
CNTN2	contactin 2 (axonal)	0.452	4.15E-4
ATP2B2	ATPase, Ca++ transporting, plasma membrane 2	0.452	4.15E-4
DNAH5	dynein, axonemal, heavy chain 5	0.453	4.10E-4
LRTM1	leucine-rich repeats and transmembrane domains 1	0.453	4.07E-4
C6orf81	chromosome 6 open reading frame 81	0.453	4.07E-4
C7orf51	chromosome 7 open reading frame 51	0.453	4.07E-4
GGT5	gamma-glutamyltransferase 5	0.453	4.06E-4
LGI2	leucine-rich repeat LGI family, member 2	0.453	4.06E-4
NKD2	naked cuticle homolog 2 (Drosophila)	0.453	4.03E-4
PCDH10	protocadherin 10	0.453	4.03E-4
KLHDC7A	kelch domain containing 7A	0.453	4.00E-4
SCNN1B	sodium channel, nonvoltage-gated 1, beta	0.454	3.97E-4
BAI1	brain-specific angiogenesis inhibitor 1	0.454	3.97E-4
PRSS8	protease, serine, 8	0.454	3.97E-4
CDH13	cadherin 13, H-cadherin (heart)	0.454	3.94E-4
PKP1	plakophilin 1 (ectodermal dysplasia/skin fragility syndrome)	0.454	3.91E-4
OR6W1P	olfactory receptor, family 6, subfamily W, member 1 pseudogene	0.454	3.91E-4
MB	myoglobin	0.454	3.91E-4

Gene symbol	Gene title	ρ	Adjusted P
TRIM15	tripartite motif-containing 15	0.455	3.86E-4
LAMA4	laminin, alpha 4	0.455	3.86E-4
GUCA2A	guanylate cyclase activator 2A (guanylin)	0.455	3.84E-4
KLF9	Kruppel-like factor 9	0.456	3.72E-4
CNTNAP5	contactin associated protein-like 5	0.456	3.72E-4
FOXM1	forkhead box M1	0.456	3.69E-4
ADCY6	adenylate cyclase 6	0.456	3.69E-4
RUFY4	RUN and FYVE domain containing 4	0.456	3.67E-4
EGFR	epidermal growth factor receptor	0.456	3.67E-4
GREB1	growth regulation by estrogen in breast cancer 1	0.456	3.67E-4
PAQR6	progestin and adipoQ receptor family member VI	0.457	3.65E-4
TMEM146	transmembrane protein 146	0.457	3.65E-4
PIGR	polymeric immunoglobulin receptor	0.457	3.62E-4
HTRA3	HtrA serine peptidase 3	0.457	3.58E-4
C5orf38	chromosome 5 open reading frame 38	0.458	3.56E-4
TAS1R1	taste receptor, type 1, member 1	0.458	3.56E-4
RIMS2	regulating synaptic membrane exocytosis 2	0.458	3.55E-4
SLC22A2	solute carrier family 22 (organic cation transporter), member 2	0.458	3.55E-4
HSPA12A	heat shock 70kDa protein 12A	0.458	3.53E-4
LRRTM1	leucine rich repeat transmembrane neuronal 1	0.458	3.53E-4
GLI2	GLI family zinc finger 2	0.458	3.53E-4
B4GALNT4	beta-1,4-N-acetyl-galactosaminyl transferase 4	0.458	3.52E-4
ABCG5	ATP-binding cassette, sub-family G (WHITE), member 5	0.458	3.50E-4
MIOX	myo-inositol oxygenase	0.458	3.50E-4
ARL4A	ADP-ribosylation factor-like 4A	0.459	3.48E-4
PAX8	paired box 8	0.459	3.44E-4
CCDC129	coiled-coil domain containing 129	0.459	3.44E-4
ADRB1	adrenergic, beta-1-, receptor	0.459	3.44E-4
FCN3	ficolin (collagen/fibrinogen domain containing) 3 (Hakata antigen)	0.459	3.42E-4
PRDM13	PR domain containing 13	0.459	3.40E-4
TERT	telomerase reverse transcriptase	0.459	3.40E-4
CHTF18	CTF18, chromosome transmission fidelity factor 18 homolog (S. cerevisiae)	0.460	3.39E-4
ATP10B	ATPase, class V, type 10B	0.460	3.39E-4
IGFN1	immunoglobulin-like and fibronectin type III domain containing 1	0.460	3.36E-4
TMEM196	transmembrane protein 196	0.460	3.34E-4
BPESC1	blepharophimosis, epicanthus inversus and ptosis, candidate 1 (non-protein coding)	0.460	3.32E-4
PPL	periplakin	0.460	3.32E-4
PRR15	proline rich 15	0.461	3.31E-4
SYT5	synaptotagmin V	0.461	3.31E-4
SLC2A5	solute carrier family 2 (facilitated glucose/fructose transporter), member 5	0.461	3.31E-4
KCNK5	potassium channel, subfamily K, member 5	0.461	3.29E-4
GRID2	glutamate receptor, ionotropic, delta 2	0.461	3.27E-4
GPD1	glycerol-3-phosphate dehydrogenase 1 (soluble)	0.461	3.27E-4
C6orf141	chromosome 6 open reading frame 141	0.461	3.27E-4

Gene symbol	Gene title	ρ	Adjusted P
COL12A1	collagen, type XII, alpha 1	0.461	3.24E-4
TDRD10	tudor domain containing 10	0.461	3.24E-4
EGFLAM	EGF-like, fibronectin type III and laminin G domains	0.462	3.22E-4
MYCN	v-myc myelocytomatosis viral related oncogene, neuroblastoma derived (avian)	0.462	3.22E-4
TTC25	tetratricopeptide repeat domain 25	0.462	3.13E-4
C1orf175	chromosome 1 open reading frame 175	0.463	3.11E-4
ADORA2B	adenosine A2b receptor	0.463	3.09E-4
EPB41L4B	erythrocyte membrane protein band 4.1 like 4B	0.463	3.08E-4
TFR2	transferrin receptor 2	0.463	3.06E-4
CLDN11	claudin 11	0.463	3.04E-4
SLC25A18	solute carrier family 25 (mitochondrial carrier), member 18	0.463	3.04E-4
ADORA3	adenosine A3 receptor	0.463	3.04E-4
PTCH2	patched 2	0.463	3.04E-4
CHRD	chordin	0.464	3.03E-4
RAPGEF4	Rap guanine nucleotide exchange factor (GEF) 4	0.464	3.03E-4
NPNT	nephronectin	0.464	2.96E-4
FAM3D	family with sequence similarity 3, member D	0.465	2.95E-4
GRM1	glutamate receptor, metabotropic 1	0.465	2.89E-4
ADCYAP1R1	adenylate cyclase activating polypeptide 1 (pituitary) receptor type I	0.466	2.88E-4
SRCIN1	SRC kinase signaling inhibitor 1	0.466	2.88E-4
TBX2	T-box 2	0.466	2.88E-4
PTPRU	protein tyrosine phosphatase, receptor type, U	0.466	2.88E-4
NEK2	NIMA (never in mitosis gene a)-related kinase 2	0.466	2.87E-4
FAM46B	family with sequence similarity 46, member B	0.466	2.83E-4
ADORA1	adenosine A1 receptor	0.467	2.80E-4
SLC6A7	solute carrier family 6 (neurotransmitter transporter, L-proline), member 7	0.467	2.78E-4
SHH	sonic hedgehog	0.467	2.76E-4
COL1A2	collagen, type I, alpha 2	0.467	2.75E-4
FAM83A	family with sequence similarity 83, member A	0.467	2.75E-4
ACHE	acetylcholinesterase	0.467	2.75E-4
FBXL7	F-box and leucine-rich repeat protein 7	0.468	2.72E-4
SLC22A13	solute carrier family 22 (organic anion transporter), member 13	0.468	2.71E-4
DCST1	DC-STAMP domain containing 1	0.468	2.71E-4
GTSE1	G-2 and S-phase expressed 1	0.468	2.71E-4
EFNA1	ephrin-A1	0.469	2.66E-4
PER1	period homolog 1 (Drosophila)	0.469	2.66E-4
CORIN	corin, serine peptidase	0.469	2.64E-4
TMC3	transmembrane channel-like 3	0.469	2.63E-4
GPC2	glypican 2	0.469	2.61E-4
ALOX15B	arachidonate 15-lipoxygenase, type B	0.469	2.61E-4
CDC20B	cell division cycle 20 homolog B (S. cerevisiae)	0.469	2.59E-4
ACOXL	acyl-CoA oxidase-like	0.469	2.59E-4
TMEM130	transmembrane protein 130	0.470	2.50E-4
WWC1	WW and C2 domain containing 1	0.470	2.50E-4
AGXT2	alanine--glyoxylate aminotransferase 2	0.471	2.49E-4

Gene symbol	Gene title	ρ	Adjusted P
CRYGA	crystallin, gamma A	0.471	2.49E-4
SLC44A3	solute carrier family 44, member 3	0.471	2.48E-4
ROR2	receptor tyrosine kinase-like orphan receptor 2	0.471	2.48E-4
SLC34A2	solute carrier family 34 (sodium phosphate), member 2	0.471	2.48E-4
MYBPH	myosin binding protein H	0.471	2.47E-4
BRSK2	BR serine/threonine kinase 2	0.471	2.45E-4
C3orf27	chromosome 3 open reading frame 27	0.472	2.40E-4
LYPD5	LY6/PLAUR domain containing 5	0.472	2.39E-4
MFAP2	microfibrillar-associated protein 2	0.472	2.37E-4
KIF1A	kinesin family member 1A	0.472	2.37E-4
ESPL1	extra spindle pole bodies homolog 1 (S. cerevisiae)	0.473	2.35E-4
SEMA3B	sema domain, immunoglobulin domain (Ig), short basic domain, secreted, (semaphorin) 3B	0.473	2.34E-4
GALNTL2	UDP-N-acetyl-alpha-D-galactosamine:polypeptide N-acetylgalactosaminyltransferase-like 2	0.473	2.34E-4
JAG2	jagged 2	0.473	2.33E-4
NGEF	neuronal guanine nucleotide exchange factor	0.473	2.30E-4
WNT2	wingless-type MMTV integration site family member 2	0.473	2.30E-4
ADCY8	adenylate cyclase 8 (brain)	0.473	2.30E-4
TMIE	transmembrane inner ear	0.474	2.29E-4
ETV3L	ets variant 3-like	0.474	2.29E-4
CADPS	Ca++-dependent secretion activator	0.474	2.27E-4
CRB1	crumbs homolog 1 (Drosophila)	0.474	2.26E-4
C20orf26	chromosome 20 open reading frame 26	0.474	2.26E-4
VSTM2A	V-set and transmembrane domain containing 2A	0.474	2.26E-4
ZYG11A	zyg-11 homolog A (C. elegans)	0.474	2.26E-4
CRYAA	crystallin, alpha A	0.474	2.23E-4
PRSS50	protease, serine, 50	0.475	2.22E-4
B4GALNT1	beta-1,4-N-acetyl-galactosaminyl transferase 1	0.475	2.22E-4
HEATR7B2	HEAT repeat family member 7B2	0.475	2.20E-4
PRSS55	protease, serine, 55	0.475	2.18E-4
OSBPL6	oxysterol binding protein-like 6	0.475	2.15E-4
SPOCD1	SPOC domain containing 1	0.476	2.14E-4
C1orf157	chromosome 1 open reading frame 157	0.476	2.14E-4
TMEM184A	transmembrane protein 184A	0.476	2.13E-4
GALNT14	UDP-N-acetyl-alpha-D-galactosamine:polypeptide N-acetylgalactosaminyltransferase 14 (GalNAc-T14)	0.476	2.11E-4
ENTPD8	ectonucleoside triphosphate diphosphohydrolase 8	0.476	2.11E-4
SLC6A1	solute carrier family 6 (neurotransmitter transporter, GABA), member 1	0.476	2.10E-4
RADIL	Ras association and DIL domains	0.476	2.10E-4
ZAN	zonadhesin	0.477	2.09E-4
KCNA5	potassium voltage-gated channel, shaker-related subfamily, member 5	0.477	2.03E-4
CDHR2	cadherin-related family member 2	0.477	2.03E-4
TMPRSS9	transmembrane protease, serine 9	0.478	2.01E-4
YPEL4	yippee-like 4 (Drosophila)	0.478	2.01E-4
GRM6	glutamate receptor, metabotropic 6	0.478	2.01E-4
SLC2A7	solute carrier family 2 (facilitated glucose transporter),	0.478	2.00E-4

Gene symbol	Gene title	ρ	Adjusted *P*
	member 7		
COL5A2	collagen, type V, alpha 2	0.478	2.00E-4
TNR	tenascin R (restrictin, janusin)	0.478	1.99E-4
CDKN2B	cyclin-dependent kinase inhibitor 2B (p15, inhibits CDK4)	0.479	1.98E-4
FGD5	FYVE, RhoGEF and PH domain containing 5	0.479	1.97E-4
CRNN	cornulin	0.479	1.97E-4
PAPPA2	pappalysin 2	0.479	1.97E-4
PADI6	peptidyl arginine deiminase, type VI	0.479	1.94E-4
EPS8L2	EPS8-like 2	0.479	1.93E-4
PRTN3	proteinase 3	0.480	1.92E-4
BTNL2	butyrophilin-like 2 (MHC class II associated)	0.480	1.89E-4
CRMP1	collapsin response mediator protein 1	0.481	1.86E-4
ADCY5	adenylate cyclase 5	0.481	1.86E-4
SLC7A14	solute carrier family 7 (cationic amino acid transporter, y+ system), member 14	0.481	1.85E-4
SLC26A9	solute carrier family 26, member 9	0.481	1.85E-4
SEMA5B	sema domain, seven thrombospondin repeats (type 1 and type 1-like), transmembrane domain (TM) and short cytoplasmic domain, (semaphorin) 5B	0.481	1.83E-4
ADAMTS9	ADAM metallopeptidase with thrombospondin type 1 motif, 9	0.482	1.79E-4
LPHN2	latrophilin 2	0.482	1.79E-4
MIB2	mindbomb homolog 2 (Drosophila)	0.482	1.79E-4
COL4A1	collagen, type IV, alpha 1	0.483	1.76E-4
GPR116	G protein-coupled receptor 116	0.483	1.73E-4
E2F8	E2F transcription factor 8	0.484	1.71E-4
SLC6A11	solute carrier family 6 (neurotransmitter transporter, GABA), member 11	0.484	1.68E-4
PTPRF	protein tyrosine phosphatase, receptor type, F	0.485	1.66E-4
LACRT	lacritin	0.485	1.65E-4
ABCA4	ATP-binding cassette, sub-family A (ABC1), member 4	0.485	1.64E-4
CAPN9	calpain 9	0.486	1.60E-4
ROBO1	roundabout, axon guidance receptor, homolog 1 (Drosophila)	0.487	1.58E-4
TMEM37	transmembrane protein 37	0.487	1.58E-4
COL23A1	collagen, type XXIII, alpha 1	0.487	1.57E-4
SDK1	sidekick homolog 1, cell adhesion molecule (chicken)	0.487	1.55E-4
C17orf73	chromosome 17 open reading frame 73	0.487	1.55E-4
TRIM31	tripartite motif-containing 31	0.488	1.55E-4
MYT1L	myelin transcription factor 1-like	0.488	1.54E-4
ELN	elastin	0.488	1.52E-4
TLL2	tolloid-like 2	0.488	1.52E-4
SOSTDC1	sclerostin domain containing 1	0.489	1.50E-4
CRHR2	corticotropin releasing hormone receptor 2	0.489	1.49E-4
PKHD1	polycystic kidney and hepatic disease 1 (autosomal recessive)	0.489	1.48E-4
ELANE	elastase, neutrophil expressed	0.491	1.42E-4
NES	nestin	0.491	1.40E-4
FIBCD1	fibrinogen C domain containing 1	0.492	1.39E-4
TEKT2	tektin 2 (testicular)	0.492	1.39E-4
TPBG	trophoblast glycoprotein	0.492	1.39E-4
NFASC	neurofascin	0.492	1.38E-4

Gene symbol	Gene title	ρ	Adjusted *P*
SORCS2	sortilin-related VPS10 domain containing receptor 2	0.492	1.38E-4
BATF2	basic leucine zipper transcription factor, ATF-like 2	0.493	1.37E-4
PLEKHN1	pleckstrin homology domain containing, family N member 1	0.493	1.36E-4
ANO7	anoctamin 7	0.493	1.33E-4
EGR4	early growth response 4	0.494	1.33E-4
FRAS1	Fraser syndrome 1	0.494	1.30E-4
GPER	G protein-coupled estrogen receptor 1	0.495	1.25E-4
EFEMP1	EGF-containing fibulin-like extracellular matrix protein 1	0.496	1.25E-4
MOCS1	molybdenum cofactor synthesis 1	0.496	1.25E-4
GAD1	glutamate decarboxylase 1 (brain, 67kDa)	0.496	1.25E-4
KLK3	kallikrein-related peptidase 3	0.496	1.24E-4
GPR113	G protein-coupled receptor 113	0.496	1.24E-4
HBEGF	heparin-binding EGF-like growth factor	0.496	1.24E-4
ZDHHC1	zinc finger, DHHC-type containing 1	0.496	1.24E-4
BOC	Boc homolog (mouse)	0.497	1.23E-4
FN1	fibronectin 1	0.497	1.22E-4
TGM2	transglutaminase 2 (C polypeptide, protein-glutamine-gamma-glutamyltransferase)	0.498	1.19E-4
IGDCC3	immunoglobulin superfamily, DCC subclass, member 3	0.498	1.19E-4
RALYL	RALY RNA binding protein-like	0.498	1.18E-4
SLC7A2	solute carrier family 7 (cationic amino acid transporter, y+ system), member 2	0.499	1.14E-4
TBR1	T-box, brain, 1	0.499	1.12E-4
FBXL2	F-box and leucine-rich repeat protein 2	0.501	1.09E-4
ZIC4	Zic family member 4	0.502	1.06E-4
COL9A2	collagen, type IX, alpha 2	0.502	1.05E-4
AREG	amphiregulin	0.502	1.04E-4
IL17RC	interleukin 17 receptor C	0.503	1.01E-4
AIM1L	absent in melanoma 1-like	0.503	1.01E-4
CCDC24	coiled-coil domain containing 24	0.503	1.00E-4
NGB	neuroglobin	0.503	1.00E-4
CELSR2	cadherin, EGF LAG seven-pass G-type receptor 2 (flamingo homolog, Drosophila)	0.505	9.42E-5
NOVA2	neuro-oncological ventral antigen 2	0.505	9.33E-5
CKM	creatine kinase, muscle	0.506	9.23E-5
SVOPL	SVOP-like	0.506	9.23E-5
MEGF10	multiple EGF-like-domains 10	0.506	9.17E-5
TIMP4	TIMP metallopeptidase inhibitor 4	0.506	9.14E-5
SEPT12	septin 12	0.506	9.13E-5
KCNMA1	potassium large conductance calcium-activated channel, subfamily M, alpha member 1	0.507	9.03E-5
LAMB4	laminin, beta 4	0.507	9.00E-5
PDZD2	PDZ domain containing 2	0.507	9.00E-5
C1orf228	chromosome 1 open reading frame 228	0.507	9.00E-5
BCAN	brevican	0.507	9.00E-5
HEYL	hairy/enhancer-of-split related with YRPW motif-like	0.508	8.75E-5
SP5	Sp5 transcription factor	0.509	8.56E-5
GRB10	growth factor receptor-bound protein 10	0.511	7.92E-5
CACNA1S	calcium channel, voltage-dependent, L type, alpha 1S subunit	0.511	7.92E-5

Gene symbol	Gene title	ρ	Adjusted P
SCN5A	sodium channel, voltage-gated, type V, alpha subunit	0.511	7.90E-5
MAGI2	membrane associated guanylate kinase, WW and PDZ domain containing 2	0.511	7.90E-5
TTC23L	tetratricopeptide repeat domain 23-like	0.511	7.89E-5
CELF3	CUGBP, Elav-like family member 3	0.511	7.89E-5
OPN5	opsin 5	0.511	7.89E-5
NUDT16	nudix (nucleoside diphosphate linked moiety X)-type motif 16	0.513	7.48E-5
CENPA	centromere protein A	0.514	7.37E-5
CHAT	choline O-acetyltransferase	0.514	7.37E-5
SLC6A3	solute carrier family 6 (neurotransmitter transporter, dopamine), member 3	0.515	6.98E-5
PCDH17	protocadherin 17	0.516	6.79E-5
DOK7	docking protein 7	0.517	6.57E-5
MTMR9LP	myotubularin related protein 9-like, pseudogene	0.518	6.49E-5
CLCN1	chloride channel 1, skeletal muscle	0.518	6.45E-5
UGT3A2	UDP glycosyltransferase 3 family, polypeptide A2	0.521	6.07E-5
GCKR	glucokinase (hexokinase 4) regulator	0.521	6.04E-5
HSD3B7	hydroxy-delta-5-steroid dehydrogenase, 3 beta- and steroid delta-isomerase 7	0.522	5.94E-5
KDR	kinase insert domain receptor (a type III receptor tyrosine kinase)	0.523	5.83E-5
GATA4	GATA binding protein 4	0.523	5.80E-5
GCK	glucokinase (hexokinase 4)	0.523	5.76E-5
B4GALT2	UDP-Gal:betaGlcNAc beta 1,4- galactosyltransferase, polypeptide 2	0.524	5.60E-5
TPPP	tubulin polymerization promoting protein	0.526	5.22E-5
MYO10	myosin X	0.527	5.15E-5
SLC22A14	solute carrier family 22, member 14	0.528	5.03E-5
FLNC	filamin C, gamma	0.529	4.74E-5
KIF25	kinesin family member 25	0.530	4.70E-5
ABI3BP	ABI family, member 3 (NESH) binding protein	0.530	4.66E-5
SLC1A3	solute carrier family 1 (glial high affinity glutamate transporter), member 3	0.530	4.62E-5
ESRRG	estrogen-related receptor gamma	0.531	4.51E-5
TMEM108	transmembrane protein 108	0.532	4.50E-5
PRKAG3	protein kinase, AMP-activated, gamma 3 non-catalytic subunit	0.532	4.48E-5
CLDN18	claudin 18	0.533	4.31E-5
SLC9A3	solute carrier family 9 (sodium/hydrogen exchanger), member 3	0.533	4.30E-5
COL16A1	collagen, type XVI, alpha 1	0.534	4.30E-5
CRISP2	cysteine-rich secretory protein 2	0.535	4.15E-5
CADM3	cell adhesion molecule 3	0.536	4.12E-5
THBS2	thrombospondin 2	0.537	3.97E-5
FGFR3	fibroblast growth factor receptor 3	0.538	3.90E-5
PADI3	peptidyl arginine deiminase, type III	0.539	3.73E-5
LRP2	low density lipoprotein receptor-related protein 2	0.541	3.58E-5
PGLYRP4	peptidoglycan recognition protein 4	0.542	3.50E-5
SH3RF2	SH3 domain containing ring finger 2	0.543	3.41E-5
SCGN	secretagogin, EF-hand calcium binding protein	0.544	3.27E-5

Gene symbol	Gene title	ρ	Adjusted *P*
ATP2C2	ATPase, Ca++ transporting, type 2C, member 2	0.544	3.27E-5
VSX1	visual system homeobox 1	0.545	3.16E-5
ANKRD22	ankyrin repeat domain 22	0.546	3.16E-5
CFB	complement factor B	0.546	3.16E-5
KIF17	kinesin family member 17	0.550	2.78E-5
MICALL2	MICAL-like 2	0.551	2.77E-5
ECE2	endothelin converting enzyme 2	0.551	2.71E-5
KLB	klotho beta	0.554	2.49E-5
IQGAP3	IQ motif containing GTPase activating protein 3	0.555	2.46E-5
C8A	complement component 8, alpha polypeptide	0.555	2.46E-5
AMPH	amphiphysin	0.561	1.97E-5
MATN2	matrilin 2	0.575	1.47E-5
HJURP	Holliday junction recognition protein	0.590	8.27E-6
ADAMTS2	ADAM metallopeptidase with thrombospondin type 1 motif, 2	0.609	3.78E-6
OPRD1	opioid receptor, delta 1	0.616	3.41E-6

Note – ρ is the Spearman's rank correlation coefficient. *P*-values were calculated by Spearman's rank correlation test between protein-coding gene expression level and sarcoidosis severity and adjusted by Benjamini & Hochberg procedure.

Table S3. Sarcoidosis clinically defined phenotype

Complicated
Lung involvement: Documented parenchymal lung disease by CT scan, radiographic stages III and IV and/or PFT with FVC <50%.<br>Organ involvement: Cardiac or neurological involvement in addition to the lungs.
Uncomplicated
Skin or pulmonary involvement limited to the mediastinal lymphadenopathy documented by CT or radiographic stages I and II.

Table S4. Patient characteristics and medications

Characteristics	Uncomplicated cases (n=17)	Complicated cases (n=22)
Gender (Female/Male)	12/5	17/5
Age (mean/sd)	47 ± 6	48 ±11
FVC<50% (n)	0	8
Neurologic (n)	0	5
Cardiac (n)	0	4
Mycophenolate (n)	1	3
Corticosteroids (n)	7	11
Hydroxychoroloquine (n)	0	1
Anti-TNF alpha therapy	0	3
